# Can Thinning Foster Forest Genetic Adaptation to Drought? A Demo‐Genetic Modelling Approach With Disturbance Regimes

**DOI:** 10.1111/eva.70051

**Published:** 2024-12-09

**Authors:** Victor Fririon, Hendrik Davi, Sylvie Oddou‐Muratorio, Gauthier Ligot, François Lefèvre

**Affiliations:** ^1^ INRAE, UR 629 Ecologie des Forêts Méditerranéennes (URFM), Domaine Saint Paul–Site Agroparc Avignon France; ^2^ INRAE, UMR 1224 Ecologie Comportementale et Biologie des Populations de Poissons (ECOBIOP), Aquapôle Saint‐Pée‐sur‐Nivelle France; ^3^ University of Liège, Gembloux Agro‐Bio Tech, TERRA, ForestIsLife Gembloux Belgium

**Keywords:** demo‐genetic modelling, disturbance regimes, drought, eco‐evolutionary dynamics, evolution‐oriented forestry, genetic adaptation

## Abstract

In managed populations—whether for production or conservation—management practices can interfere with natural eco‐evolutionary processes, providing opportunities to mitigate immediate impacts of disturbances or enhance selection on tolerance traits. Here, we used a modelling approach to explore the interplay and feedback loops among drought regimes, natural selection and tree thinning in naturally regenerated monospecific forests. We conducted a simulation experiment spanning three nonoverlapping generations with the individual‐based demo‐genetic model Luberon2. Luberon2 integrates forest dynamics processes driving survival and mating success, including tree growth, competition, drought impacts and regeneration, with genetic variation in quantitative traits related to these processes. We focused on two variable traits: individual vigour, determining diameter growth potential without stress as the deviation from average stand growth, and individual sensitivity to drought stress as the slope of the relationship between diameter growth and drought stress level. We simulated simplified thinning scenarios, tailored to even‐aged stands. Considering plausible genetic variation and contrasting drought regimes, the predicted evolutionary rates for both traits aligned with documented rates in wild plant and animal populations. Thinning considerably reduced natural selective pressures caused by competition and drought compared to unthinned stands. However, the conventional thinning practice of retaining the larger trees resulted in indirect anthropogenic selection that enhanced genetic gain in vigour and lowered sensitivity by up to 30%. More intensive thinning aimed at reducing drought stress by reducing stand density hampered the selection against sensitivity to drought, potentially hindering long‐term adaptation. Conversely, avoiding the early, nonselective thinning step—thereby promoting both natural and anthropogenic selection—ultimately resulted in better stand performance while maintaining long‐term evolvability. This study emphasises the potential of evolution‐oriented forestry strategies to combine drought stress mitigation with genetic adaptation. It provides general insights into how population management, disturbance regimes and eco‐evolutionary responses interfere, aiding sustainable decision‐making amid environmental uncertainties.

## 1 Introduction

Populations can adapt to repeated disturbances within and across generations through various mechanisms: Population rearrangements (e.g., demographic or spatial structure), individual‐level acclimation (which relates to adaptive plasticity) and/or genetic evolution (Alfaro et al. 2014; Moran et al. 2017; Sergio, Blas, and Hiraldo 2018; Gloy, Herzschuh, and Kruse 2023). The disturbance regime characterises the type, frequency and intensity of disturbances occurring at a specific location (White and Jentsch 2001; Banks et al. 2013). When disturbances are frequent and predictable in an otherwise long‐term stable environment, local adaptation*^1^ can occur, contingent on sufficient genetic variation (Lytle 2001; Tonnabel et al. 2012; Malíková et al. 2016; Bansal, Harrington, and St. Clair 2016). Disturbance regimes not only determine the expression of adaptive genetic variation in traits driving the individual fitness* components (growth, survival and reproduction) but they also drive the intensity of hard and soft selection processes* and, ultimately, the eco‐evolutionary dynamics* of population vulnerability. In managed populations for production or conservation purposes, management practices have multifaceted effects on the eco‐evolutionary dynamics, including influencing the vulnerability or exposure to disturbances. Understanding the interplay among disturbance regimes, evolutionary processes and management practices is fundamental for developing strategies that promote population resilience—referring here to the ability of a population to recover and maintain its essential functions and structure after disturbance.

In the context of forestry, and more particularly within natural regeneration forest systems considered in this work, disturbance regimes are one of the key parameters to account for in forest management* and silviculture strategies*, which overall aim at choosing locally appropriate tending and regeneration methods to sustainably achieve multiple objectives (Achim et al. 2022). One lever of action involves shaping the composition and structure of forest tree populations to mitigate direct disturbance impacts (Jactel et al. 2009; Moreau et al. 2022). The silvicultural systems (Nyland et al. 2016) integrate multiple management practices, including the choice of species to plant or favour, thinning* and the types of regeneration*—whether natural or artificial—which are key determinants of the structure and composition of managed forest stands. Forest composition and structure, in turn, influence the stress level concomitant with various biotic and abiotic disturbances and/or the probability of their occurrence. In this context, we use the term ‘stress’ as the outcome of a disturbance that is directly perceived by trees. The influence of forest management on the direct impacts of disturbances is well documented for pathogens (Tainter and Baker 1996), insect pests (Wainhouse 2005), wildfires (Peterson et al. 2005), wind gusts (Schelhaas 2008) and droughts (Metz et al. 2016). Another lever of action entails the implementation of silvicultural practices to guide the evolutionary trajectory of tree populations towards improved adaptedness, resilience and long‐term evolvability: This is known as evolution‐oriented forestry* (Lefèvre et al. 2014). The balance between the ecological benefits of stress reduction strategies and their potential evolutionary consequences on genetic adaptation or acclimation can go in a number of ways. Therefore, it is crucial to consider all these effects together to rationalise forest management strategies that support both short‐term ecological health and long‐term adaptive capacity.

Silvicultural interventions, such as pruning or thinning, can significantly decrease canopy leaf area, leading to reduced transpiration and rainwater interception and thereby alleviating drought stress (Bréda, Granier, and Aussenac 1995; Forrester et al. 2012; Duncker et al. 2012). After these interventions, the remaining trees grow under relaxed competition and gradually restore stand canopy (Chianucci et al. 2016; Tague and Moritz 2019). Hence, a common approach to reducing stand vulnerability to drought consists of more frequent and intensive thinnings maintaining low stand density that, in the short‐term, stimulates the growth of the retained trees and reduces drought‐induced mortality (Sohn, Hartig, et al. 2016; Schmitt et al. 2020; Gavinet et al. 2020; Moreau et al. 2022). However, this strategy prioritises short‐term benefits while neglecting evolutionary processes and genetic impacts (Legay et al. 2015).

Predicting the short‐term (over life cycle) and long‐term (across multiple generations) evolutionary consequences of management strategies is a challenge for long‐lived species such as trees. The demo‐genetic agent‐based modelling framework* (DG‐ABM) is well suited for developing simulation approaches that consider the intricate interplay between human interventions and evolutionary processes (Oddou‐Muratorio, Davi, and Lefèvre 2020; Lamarins et al. 2022). In line with this approach, we developed a demo‐genetic forest model, Luberon2, to investigate the joint effects of silvicultural practices and disturbance regimes on eco‐evolutionary processes within monospecific forest stands, originally those of *Cedrus atlantica* (Godineau et al. 2023). The model includes tree growth, competition and reproductive processes that dynamically drive individual fitness components such as survival and mating success. Crucially, the model leverages the quantitative genetics framework* to simulate genetic variation in traits related to individual performances. In a previous study, this model was used to quantify the intensity of competition‐induced selection for a single growth‐related trait, vigour*, which determines the radial growth potential without stress. It was shown that random thinning regimes, that is, all thinnings carried out without regard to tree size, reduced the intensity of natural selection and the evolutionary rate of vigour in the population (Godineau et al. 2023). For the present study, we extended the model to examine the interactions between more realistic thinning practices and natural eco‐evolutionary processes in an environment combining both competition and drought. To achieve this objective, we introduced customisable drought regimes into Luberon2, wherein drought stress, which is modulated by the canopy leaf area, primarily affects annual growth and potentially leads to mortality if growth falls below a threshold. In addition to vigour, we explicitly considered a second growth‐related trait, sensitivity, which modulates individual growth response to drought stress. In a previous publication, we used dendrochronological data as a proxy for vigour and sensitivity, providing empirical estimates of within‐stand phenotypic variances* and covariances (Fririon et al. 2023). Vigour was measured as the individual deviation from the stand‐level average growth, and sensitivity* as the slope of the linear relationship between individual growth and drought stress level.

We developed a working hypothesis that under certain conditions, a drought stress reduction strategy could have long‐term impacts on adaptive processes resulting from the reduction in the intensity of natural selection on sensitivity, eventually substituted by anthropogenic selection of thinned trees. Furthermore, we propose that an evolution‐oriented approach of thinning that considers both natural and anthropogenic selection processes together could foster genetic improvement over generations, potentially producing superior outcomes compared to an intensive silvicultural strategy primarily focused on drought stress reduction. To investigate these hypotheses, we conducted a simulation experiment with Luberon2 spanning three successive, nonoverlapping generations in even‐aged stands*. We used the version of the model calibrated for Douglas‐fir, *Pseudotsuga menziesii* (Mirb.) Franco, a conifer species native to western North America and extensively planted in Europe for its rapid growth and high‐quality wood. Notably sensitive to drought, Douglas‐fir responds with reduced growth and increased mortality under such conditions, prompting managers to implement preventive strategies against dieback (Sergent 2011). Our first objective was to characterise the process of natural selection on vigour and sensitivity in a scenario without silvicultural thinning, under contrasted drought stress regimes. We considered vigour and sensitivity as independent traits with distinct genetic architectures* to investigate whether a genetic correlation* between these traits might arise from the demo‐genetic dynamics of the model. We compared the evolutionary rates in vigour and sensitivity predicted by the model to empirical observations from the literature. Additionally, we quantified the potential of genetic improvement through natural selection against sensitivity to mitigate the overall impacts of drought on growth and survival. Subsequently, we explored the impacts of a traditional thinning scenario, a drought stress reduction strategy and an exploratory evolution‐oriented approach to the vulnerability of populations to drought, considering their impact on the drought stress level and the evolutionary processes. We also compared the silvicultural scenarios* with regard to their overall impacts on stand performance evaluated through the average tree size at the end of the rotations*—the period between stand regeneration and final harvest*. In our simulations, thinning followed the conventional practice for production‐oriented conifer stands, including Douglas‐fir, by preferentially removing smaller trees, that is, thinning from below (Mäkinen and Isomäki 2004; Perin et al. 2016), resulting in indirect* anthropogenic selection on vigour and sensitivity.

## 2 Material and Methods

### 2.1 General Description of Luberon2

A detailed description of Luberon2 can be found in Godineau et al. (2023). We focus here on the general aspects of the model essential to fully understand the present study.

Luberon2 is an individual‐based simulation model coupling three primary modelling components: (i) Tree‐level distance independent forest dynamics models calibrated for different species, which simulate tree growth and competition‐induced mortality (self‐thinning*); (ii) models for male and female fecundity, as well as seed and pollen dispersal, which simulate the natural regeneration process; and (iii) a finite quantitative trait loci* (QTL) genetic model that simulates the phenotypic making and heredity of variable traits. In the absence of calibration for all species, we currently use the fecundity and gamete dispersal model calibrated for the Atlas cedar for all species implemented in Luberon2, including Douglas‐fir. Additionally, Luberon2 includes tools to simulate various types of thinnings as well as disturbance regimes. The simulated forest stand is subdivided into a regular grid of pixels (15 m × 15 m by default) where growth and competition‐induced mortality occur within each pixel independently of neighbouring pixels, allowing for spatial heterogeneity in stand structure. The model runs on an annual time step. Genetic evolution results mechanically from the demographic processes, without any prescribed function between the performance traits and fitness. In particular, the larger a tree, potentially linked to its genetic performances, the less it is impacted by competition and the more seeds and pollen it produces. Therefore, the growth‐related traits are under density‐dependent soft selection due to their correlation with tree size, which is directly linked to viability and fecundity selection (bigger trees survive better and reproduce more; Godineau et al. 2023). Random or selective silvicultural interventions and natural selective disturbances jointly modify the composition, structure and demographic processes with consequences on evolutionary processes.

### 2.2 Modelling Drought Stress Level and Calibration of Three Drought Stress Regimes

For this study, we integrated a stationary, stochastic, drought stress regime generator into Luberon2, which draws an annual drought stress level modulated by the stand leaf area index* (LAI), a classic variable for quantifying the amount of leaf matter in a canopy expressed by the ratio of leaf area to soil area. A Gamma distribution Γ (shape, scale) gives the probabilities of occurrence of the drought stress level values depending on the LAI, with shape and scale computed from LAI with six parameters (*a*_shape_, *b*_shape_, *c*_shape_, *a*_scale_, *b*_scale_ and *c*_scale_):

(1) $$\text{shape}=a_{\text{shape}}\times {\mathrm{LAI}}^{b_{\text{shape}}}+c_{\text{shape}}$$

(2) $$\text{scale}=a_{\text{scale}}\times {\mathrm{LAI}}^{b_{\text{scale}}}+c_{\text{scale}}$$

Each year, for the whole stand, a random value is drawn in a uniform distribution in the interval [0, 1[. Then, in each pixel, the quantile function of the Gamma distribution returns the drought stress level corresponding to the global random value and the local LAI of the pixel.

The LAI of each pixel is obtained from an allometric relationship calibrated by Smith (1993) for Douglas fir, which computes the leaf area of individual trees according to their diameter (the larger a tree, the greater its leaf area) and the stand density (the higher the density, the smaller the individual leaf area):

(3) $$\begin{array}{l} {\mathrm{LA}}_{i}=0.4781\times {{\mathrm{DBH}}_{i}}^{1.8659}\times \exp\left(-0.0829\times {\mathrm{CRD}}_{p}\right),\\ \;\text{with}\;{\mathrm{CRD}}_{p}=\frac{{\mathrm{BA}}_{p}}{{{\mathrm{QMD}}_{p}}^{b}}\; \end{array}$$

where ${\mathrm{LA}}_{i}$ is the leaf area of an individual *i*, ${\mathrm{DBH}}_{i}$ its diameter at breast height* and ${\mathrm{CRD}}_{p}$ is the Curtis' relative density of its pixel *p*. The Curtis' relative density is obtained from the basal area*, ${\mathrm{BA}}_{p}$, and the quadratic mean diameter*, ${\mathrm{QMD}}_{p}$, of the pixel and a species‐specific parameter, b, equal to 0.5 for Douglas fir (Curtis 1982). Note that we expect to underestimate the drop in LAI after thinning interventions because the allometric equation translates the drop‐in tree density into an instantaneous increase in individual leaf area, whereas in reality, several years are necessary to observe this response (Chianucci et al. 2016; Tague and Moritz 2019).

We calibrated the drought stress regime generator for three regimes that cover the range of current drought stress conditions within the European Douglas‐fir range. Using pedoclimatic data, species‐specific ecophysiological values and an LAI value, the process‐based ecophysiological model CASTANEA (Dufrêne et al. 2005; Davi and Cailleret 2017) simulates the daily soil water potential at stand level. The daily soil water potential is then accumulated annually to produce an index, stressLevel (expressed in MPa), which quantifies the annual drought stress level (Fririon et al. 2023). We ran CASTANEA between 1979 and 2008 on the 698 raster cells (resolution of 0.5° per 0.5°) of European pedoclimatic data aggregated by Petit‐Cailleux et al. (2021) that contained at least one Douglas‐fir stand (Mauri, Strona, and San‐Miguel‐Ayanz 2017). For each of these raster cells, nine simulations were performed varying the LAI value from 2 to 10, which corresponds to the LAI range typically observed in naturally regenerating Douglas‐fir stands (Smith 1993). Among the 698 raster cells, several showed *zero drought stress*. Among those with drought stress, we selected two with contrasting average stressLevel over all years and LAI values (Appendix S2: Figure S1). These represented *medium drought stress*, which only impacts growth in Luberon2 simulations according to preliminary analysis (data not shown), and *severe drought stress*, which has a more pronounced impact on growth and often leads to mortality. Regarding the medium and severe drought stress regimes, a Gamma distribution was fitted to the stressLevel time series for each LAI condition using the ‘fitdistr {MASS}’ function in R. For each of these two regimes, the six parameters linking the scale and shape parameters of the stressLevel Gamma distributions to LAI (Equations 1 and 2) were determined by nonlinear regression using the ‘nls {stats}’ function in R (Appendix S2: Table S1). As expected, LAI exhibited a substantial influence on stressLevel for both regimes (Appendix S2: Figure S2). The zero drought stress condition was simply achieved by deactivating the drought disturbance in the simulations.

### 2.3 Modelling Individual Growth Response to Drought Stress Level

#### 2.3.1 Modulating Potential Diameter Growth by Vigour and Sensitivity

Each year, the baseline annual diameter growth of a tree is computed from its initial circumference by the distance‐independent growth model for Douglas‐fir, known as GYMNOS (Ligot et al. 2023). The parameters of this nonlinear function vary dynamically with dendrometric variables* such as stand density and dominant height (height of the tallest trees) (Deleuze et al. 2004), and it was calibrated on empirical data by Perin et al. (2013, 2017). Then, this baseline prediction is adjusted using an individual additive term, vigour, which can be positive or negative (Godineau et al. 2023). In the absence of drought stress, vigour is the only trait driving growth variation among trees, allowing them to achieve their potential diameter growth.

The baseline growth models in Luberon2 did not originally account for individual phenotypic variation in their equations. Adding phenotypic variation in vigour can lead to overestimated stand‐level average growth because vigorous trees—those that grow faster than the baseline model predicts—are selectively favoured (Godineau et al. 2023). However, we demonstrated that this growth bias has a negligible impact on predicted evolutionary rates over a few generations (Appendix S4). To test this, we added an optional dynamic correction to the model to align stand‐level average growth predictions with those of the baseline growth model and compared the results with and without the correction. While the correction enhances the accuracy of growth predictions, it distorts selection dynamics, especially under disturbance regimes, thereby restricting the exploration of eco‐evolutionary feedback and evolutionary processes (see Appendix S4 for details). For this reason, we disabled the correction for this study.

Under drought stress conditions, each year and within each independent pixel, the drought stress level impacts the potential diameter growth of each tree according to its individual sensitivity. Following a previous study of within‐stand phenotypic variation in growth response to drought in five tree species, including Douglas‐fir (Fririon et al. 2023), sensitivity corresponds to the slope of the linear relationship between diameter growth and stressLevel. In Luberon2, the overall sensitivity of an individual has two components: A constant baseline value shared by all individuals, adjusted by an individual additive term that can be either positive or negative. For an individual *i*, the annual diameter growth after drought stress impact (${\text{dDstress}}_{i}$) is computed as:

(4) $$\begin{array}{l} {\text{dDstress}}_{i}={\text{dDnoStress}}_{i}\left(1-\text{StressLevel}\times \left(\text{base}\_\text{sensitivity}+\mathrm{ind}\_{\text{sensitivity}}_{i}\right)\right),\\ \;\text{with}\;\left(\text{base}\_\text{sensitivity}+\mathrm{ind}\_{\text{sensitivity}}_{i}\right)\geq 0.1\times \text{base}\_\text{sensitivity} \end{array}$$

where ${\text{dDnoStress}}_{i}$ is the annual potential growth (including vigour), StressLevel is the drought stress level of the current year, base_sensitivity is the baseline sensitivity value and $\mathrm{ind}\_{\text{sensitivity}}_{i}$ is the individual deviation from the baseline value, hereafter referred to as sensitivity for the sake of simplicity. In these simulations, the baseline sensitivity value was set to 0.002 MPa^−1^, the average of the observed phenotypic values* within Douglas‐fir stands (Fririon et al. 2023). To prevent the possibility of negative overall sensitivity values for trees displaying a substantial negative sensitivity, which implies improved growth under drought stress, we imposed a constraint to ensure that the overall sensitivity remains at a minimum of 10% of the baseline value.

In Luberon2, severe drought impact on growth can lead to mortality following the framework proposed by Bugmann (1996) in the model ForClim: A 90% reduction in diameter growth is likely to result in tree death with a probability of 36.8%. This reproduces the mechanisms by which trees that are more sensitive to drought stress in terms of growth are at higher risk of drought‐induced mortality (DeSoto et al. 2020).

#### 2.3.2 Genetic Variation in Vigour and Sensitivity

Luberon2 uses a finite‐loci quantitative genetics model to represent within‐stand phenotypic variation (further details in Godineau et al. 2023). Here, we assumed that both vigour and sensitivity were genetically controlled by 20 diallelic* QTL each. In a previous study, it was shown that different initial sets of genotypes with the same target additive genetic variance* may result in different evolutionary rates, but the effect of the initial set of genotypes fades with the number of QTL (Godineau et al. 2023). With 20 QTL, we neglected this genetic stochasticity and we used a single set of initial genotypes for each variance level. We assumed an initial zero genetic correlation between vigour and sensitivity, that is, no pleiotropy or linkage disequilibrium*. Our goal was to assess whether such correlation could emerge from correlated selection for vigour and against sensitivity without introducing a particular a priori value at initialisation.

We simulated three initial populations, each characterised by a distinct level of phenotypic variation (Table 1). The first population had *zero phenotypic variation* (named zeroVar), that is, no QTL effects and no environmental variance, as a theoretical reference situation with no evolutionary process. The second population had a *baseline additive genetic variances* (baseVA) for vigour and sensitivity derived from the average within‐population phenotypic variance observed in Douglas‐fir plantations (Fririon et al. 2023), assuming a narrow sense heritability* of *h* ^2^ = 0.3 for both traits as classically observed for these types of quantitative traits (Alberto et al. 2013; Depardieu et al. 2020). Finally, the last population had *twice the baseline additive genetic variances* (twiceVA) with the same environmental variance as the previous one, resulting in a narrow sense heritability *h* ^2^ = 0.46, aimed to quantify the effect of a marked change in the amount of genetic variation on the performance of the forest. The genetic and environmental variances of the three populations are specified in Table 1. For baseVA and twiceVA, we employed a heuristic algorithm to simulate the initial individual genotypes, targeting the specified additive genetic variances (additional details on the algorithm are provided in Godineau et al. 2023). In all cases, we set the initial mean genotypic values* of vigour and sensitivity to 0.

**TABLE 1:** Characterisation of the three initial populations simulated: additive genetic variances for vigour and sensitivity (VA(Vig) and VA(Sensi), respectively); environmental variances for vigour and sensitivity (VE(Vig) and VE(Sensi), respectively); and narrow sense heritability (*h* ^*2*^).

Population	VA(Vig)	VA(Sensi)	VE(Vig)	VE(Sensi)	*h* ^2^
zeroVar: zero phenotypic variation	0	0	0	0	—
baseVA: baseline additive genetic variances	8.0 × 10^−3^ cm^2^	5.1 × 10^−7^ MPa^−2^	1.9 × 10^−2^ cm^2^	1.2 × 10^−6^ MPa^−2^	0.3
twiceVA: twice the baseline additive genetic variances	1.6 × 10^−2^ cm^2^	1.0 × 10^−6^ MPa^−2^	1.9 × 10^−2^ cm^2^	1.2 × 10^−6^ MPa^−2^	0.46

We investigated the feedback effect of genetic evolution on drought stress impacts and population dynamics by comparing simulations with versus without phenotypic variation. In addition, by simulating two levels of genetic variation (baseline and double the baseline) we enable a comparison between theoretical predictions and the actual model outcomes. The breeder's equation*—*R = i*·*h*·*σA*—provides a theoretical reference of the expected response of a trait under direct mass selection assuming only viability selection with constant selection gradient* and selection intensity*. With these assumptions, the expected response to selection* when doubling the additive genetic variance is 1.75 times the response with the baseline variance. Compared to this value, the increase in the response to selection obtained when doubling the genetic variance in the demo‐genetic model informs us of the departure from the theoretical assumptions of the breeder's equation.

The genetic value of a new seedling for vigour and sensitivity is inherited from its parents, but its initial diameter is determined by a random draw from a probability distribution that reflects a realistic diameter range at the age of recruitment* (15 years for Douglas‐fir) (Godineau et al. 2023). As a result, the genotype only begins to affect tree diameter after the first year of growth, and the correlation between vigour or sensitivity and tree diameter increases gradually over time. In this study, the parent trees are all within the stand, with the assumption that there is no outside gene flow*. Over the timescale considered (a few generations), we neglect the occurrence and spread of mutations.

### 2.4 Description of the Silvicultural Scenarios

**FIGURE 1:**
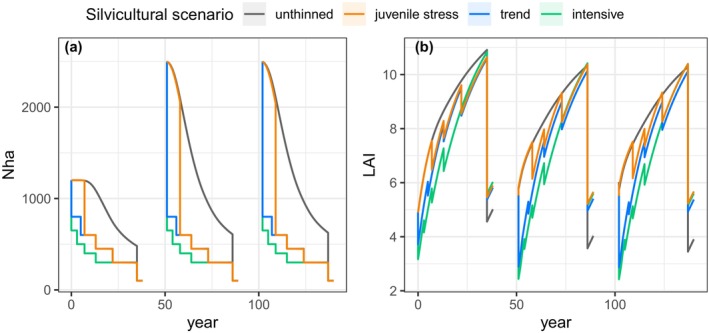
Characterisation of stand dynamics in the four different silvicultural scenarios (colours): (a) stand density, Nha; (b) leaf area index, LAI. This figure illustrates the case without phenotypic variation (zeroVar) nor drought stress; see Appendix S3: Figure S1 for the other drought stress regimes and levels of phenotypic variation. In the unthinned scenario, the decline in Nha within each rotation is only driven by competition‐related mortality, with the exception of the seeding cut and final harvest. In contrast, the other scenarios exhibit Nha changes resulting from a combination of competition‐induced mortality and thinning interventions. The LAI increases within each rotation as stand develops and decreases at each thinning intervention: The intensive scenario consistently maintains a lower LAI compared to the trend scenario, while the juvenile stress scenario maintains an LAI identical to the unthinned scenario, followed by a slight elevation above the trend scenario. During Rotations 2 and 3, a higher initial density constraints growth and tree size, leading to a lower LAI. The prerecruitment period is intentionally omitted from the representation, as the model calibration for tree density and growth begins from the recruitment age. Furthermore, trees that have not yet reached recruitment age are not considered in the Nha and the LAI.

The simulation scene had an area of 9.9225 ha (441 pixels of 15 m × 15 m). The site index*—defined in forestry as the average total height of the tallest trees at a given age—was 40 m at 50 years, corresponding to a site with high productivity for Douglas‐fir. We simulated the particular case of an initial plantation subsequently naturally regenerated. This is not only a theoretical situation: Old Douglas‐fir plantations often show favourable conditions for natural regeneration in Europe (Petit and Claessens 2013). At initialisation, trees were randomly dispersed in the scene, and new trees in the next generation were located through the seed dispersal process. We simulated three successive rotations with nonoverlapping generation turnover. The initial stand had a total number of 11,907 individual trees, corresponding to 1200 trees per hectare, that is, a classical density for Douglas‐fir plantations, while the next two rotations started with 2500 trees per hectare, that is, a plausible value for natural regeneration (Figure 1a). Each rotation lasted 50 years, as prescribed for Douglas‐fir in high fertility sites.

We simulated four silvicultural scenarios, each repeating the same sequences of interventions across successive rotations (Table 2). In all silvicultural scenarios, each rotation concluded with 3 years of regeneration triggered by an intervention known as seeding cut*, leaving 100 seed trees* per hectare. In all cases, the seeding cut was carried out randomly. At the end of these 3 years, the final harvest removed the seed trees to leave only the new seedlings. Note that the forest dynamics models were calibrated for trees above a certain age, the recruitment age (15 years for Douglas‐fir) and, before reaching this age, no process takes place in the simulations (Godineau et al. 2023). The silvicultural scenarios differed in terms of thinning regime, that is, the number, timing and intensity of thinning interventions (Table 2). We simulated an *unthinned scenario*, as a reference scenario for natural selection. In this scenario, the only interventions were the random seeding cut at 50 years, followed by the final harvest 3 years later, allowing for comparability with the other scenarios. As a baseline thinned scenario, we simulated a *trend scenario*, as proposed for Douglas‐fir by the French web service Forêts‐21 for the strategic management of planted forests (Aluome et al. 2019). This scenario entailed four thinnings. The thinnings were primarily conducted from below, selectively targeting the smallest trees, with the exception of the first thinning, which was carried out randomly, aligning with conventional forestry practices (Table 2; Perin et al. 2016). Building upon the trend scenario, we simulated two original scenarios: (i) an *intensive scenario*, characterised by earlier and more intense thinnings aimed at reducing LAI and, consequently, reducing drought stress level; and (ii) a scenario called *juvenile stress scenario* (Figure 1), where the first thinning is omitted and the second is delayed by 2 years to sustain exposure to drought stress and competition during the juvenile stage, as proposed by Fririon et al. (2023). In forestry terms, the juvenile stress scenario corresponds to a scenario without precommercial thinning*. Notably, the juvenile stress scenario entails the absence of random thinning, ensuring that no genotypes are excluded from selection processes, be it anthropogenic or natural.

**TABLE 2:** Regimes of thinning interventions in the four silvicultural scenarios. For each intervention (thinnings, seeding cut and final harvest), the age of the stand that triggers the intervention is indicated with the target number of trees per hectare (Nha) in brackets.

Scenario	Thinning 1 (random)	Thinning 2 (from below)	Thinning 3 (from below)	Thinning 4 (from below)	Seeding cut (random)	Final harvest
Unthinned	—	—	—	—	50 years (100)	53 years (0)
Trend	15 years (800)	20 years (600)	28 years (450)	37 years (300)	50 years (100)	53 years (0)
Intensive	15 years (650)	18 years (500)	22 years (400)	28 years (300)	50 years (100)	53 years (0)
Juvenile stress	—	22 years (600)	28 years (450)	37 years (300)	50 years (100)	53 years (0)

To enhance the interpretability and comparability of the simulated silvicultural scenarios, these scenarios represent a simplification of reality. In practice, the sequence of interventions and the rotation period can vary depending on the regeneration method and the initial seedling density. In our simulations, the first thinning aims to reach the same density as in the prescribed silviculture for a plantation forest. Consequently, in the second and third rotations, which result from natural regeneration, the first thinning is more severe than usual and could represent several successive thinnings in reality, potentially extending the rotation period. However, the model does not account for the potential destabilising effects of such intensive cutting on the stand, and thus it does not impact the subsequent stand evolution. Moreover, in practical forestry, seeding cuts are rarely random; instead, the best seed trees, typically the biggest ones, are selectively retained. The use of random seeding cuts in our simulations enables comparison with the unthinned scenario, where only natural selection operates. Nonetheless, a weak impact on evolutionary rate is expected, as most of the selection process has already occurred by the time the seeding cut is applied (Godineau et al. 2023).

### 2.5 Simulation Plan and Analysis of Simulation Outputs

The full factorial design, which combined three stochastic drought stress regimes, three levels of phenotypic variation and four silvicultural scenarios, resulted in 36 modalities. Each modality was replicated 10 times to account for the model's inherent stochasticity, including drought stress regimes, demographic processes and thinning interventions, leading to 360 simulations in total.

For each simulation run, we annually computed demographic, dendrometric and genetic variables at stand level (Table 3). We focused on this set of variables to characterise the effects of various combinations of thinning and drought stress regimes on: (i) the genetic evolution of vigour and sensitivity, (ii) the evolution of stand growth performance and (iii) the demographic dynamics driven by the different causes of mortality.

**TABLE 3:** Description of the demographic, dendrometric and genetic output variables analysed.

Annual variables	Code	Unit	Description
Population genetic mean of vigour	μG.Vig	cm year^−1^	The population mean genetic value in vigour. The initial value is arbitrarily 0, then evolutionary processes, e.g., selection and genetic drift*, cause the value to evolve.
Population genetic mean of sensitivity	μG.Sensi	MPa^−1^	The population mean genetic value in sensitivity. The initial value is arbitrarily 0, then evolutionary processes, e.g., selection and genetic drift, cause the value to evolve.
Additive genetic variance of vigour	VA(Vig)	cm^2^ year^−2^	The variance of individual vigour genetic values within the population.
Additive genetic variance of sensitivity	VA(Sensi)	MPa^−2^	The variance of individual sensitivity genetic values within the population.
Genetic correlation between vigour and sensitivity	Cor (Vig, Sensi)	—	Pearson's correlation between individual genetic values of vigour and sensitivity. The initial value is 0. Then, selection and drift drive the evolution of this value.
Quadratic mean diameter	QMD	cm	The geometric/quadratic mean of tree diameter, a standard measure for characterising stand development stages (Curtis and Marshall 2000), is computed for the entire stand.
Stand density	Nha	—	The number of living and recruited trees (older than 15 years) per hectare.
Competition‐induced mortality rate	CMR	—	The ratio between the cumulative number of deaths by self‐thinning and the number of initial trees. It is computed separately for each rotation.
Drought stress‐induced mortality rate	SMR	—	The ratio between the cumulative number of deaths by severe drought stress impact on growth and the number of initial trees. It is computed separately for each rotation.

Following Godineau et al. (2023), we computed the average evolutionary rate per generation for vigour and sensitivity, $H_{0}$, defined by Gingerich (1993, 2009). Here, we were interested in the overall magnitude of change in vigour and sensitivity rather than its direction, which is inherently deterministic. We therefore expressed $H_{0}$ in absolute value:

(5) $$H_{0}=\left|\left({\text{zdiff}}_{i}/\sqrt{\text{zvar}.w_{i}}\right)/n.\mathrm{gen}\right|$$

where ${\text{zdiff}}_{i}$ is the observed change in the mean value of the trait in a temporal interval *i*, $\text{zvar}.w_{i}$ is the pooled within‐population phenotypic variance of the trait sampled at the beginning and at the end of the temporal interval *i*, and n.gen is the number of generations. The temporal interval that we used is defined by the beginning of the first rotation and the regeneration produced after the third, corresponding to three complete generations.

## 3 Results

### 3.1 Natural Selection of Vigour and Sensitivity Through Competition and Drought Stress in the Unthinned Scenario

**FIGURE 2:**
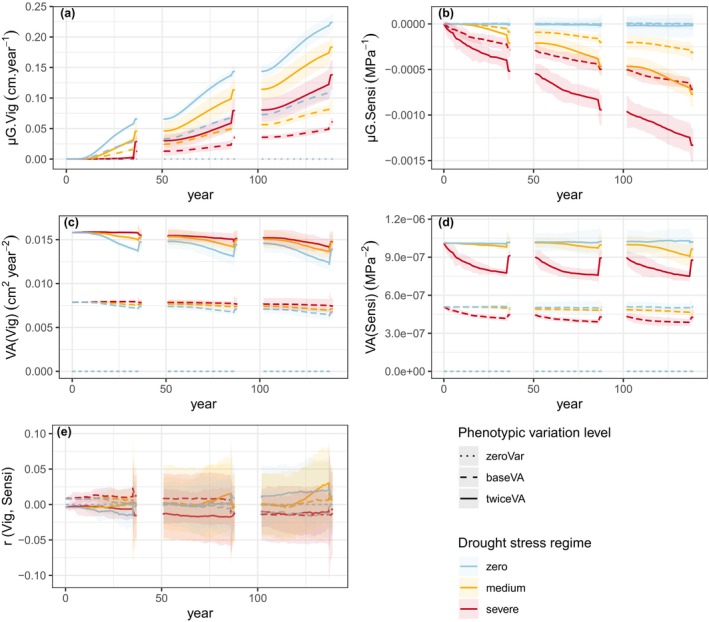
Dynamics of population genetic parameters in the unthinned scenario across three successive rotations according to the level of phenotypic variation (line type) and drought stress regime (colours): (a) Population genetic mean of vigour, μG.Vig; (b) population genetic mean of sensitivity, μG.Sensi; (c) additive genetic variance of vigour, VA(Vig); (d) additive genetic variance of sensitivity, VA(Sensi); and (e) genetic correlation between vigour and sensitivity, *r* (Vig, Sensi). In the absence of drought stress, sensitivity underwent no selective pressure; any minor changes were solely a result of genetic drift. The shaded areas represent the 95% intervals over 10 replicates for each genetic setup. The prerecruitment period is intentionally omitted from the representation, as the model does not account for any demo‐genetic changes during this phase.

In the unthinned scenario, the genetic mean of vigour continuously increased, both within and across generations (Figure 2a). The genetic mean of sensitivity remained stable in the absence of drought stress, and decreased otherwise (Figure 2b). Within each generation, changes in genetic means of both traits proceeded first through competition‐induced viability selection and then by fecundity selection during the reproduction phase when more vigorous and/or less sensitive genotypes contributed more due to their larger size, causing qualitative leaps in genetic means. Simultaneously, the genetic variances of both traits decreased under viability selection by competition (Figure 2c,d). The variances were partly restored during the reproduction phase showing that selection mainly created linkage disequilibrium between QTL without much affecting QTL allele frequencies. Under the severe drought stress regime, drought stress‐induced mortality started at the recruitment age, potentially driving the genetic evolution of sensitivity prior to any competition‐induced mortality, as evident in the first rotation (Figure 2a,b). The co‐selection for vigour and against sensitivity did not generate a clear genetic correlation between vigour and sensitivity. Only a slight positive correlation was observed with twice the baseline genetic variance (twiceVA) under the medium drought stress regime (Figure 2e).

Over three generations, a more severe drought stress regime resulted in higher genetic gain* against sensitivity but lower genetic gain for vigour, highlighting antagonistic selection processes between the two traits (Table 4).

**TABLE 4:** Genetic change in vigour (top) and sensitivity (bottom) over three generations according to the drought stress regime (columns) and initial genetic variance (lines). The corresponding evolutionary rate per generation ($H_{0}$, Equation 5) is indicated in brackets. Note that in the absence of drought stress, sensitivity underwent no selective pressure; the minor changes observed were solely a result of genetic drift, with an evolutionary rate close to 0.

	Zero drought stress	Medium drought stress	Severe drought stress
Vigour (cm year^−1^)			
baseVA	+1.1 × 10^−1^ (0.23)	+8.6 × 10^−2^ (0.18)	+6.2 × 10^−2^ (0.13)
twiceVA	+2.2 × 10^−1^ (0.41)	+1.8 × 10^−1^ (0.33)	+1.4 × 10^−1^ (0.25)
Sensitivity (MPa^−1^)			
baseVA	+5.4 × 10^−7^ (≈0)	−3.2 × 10^−4^ (0.08)	−7.2 × 10^−4^ (0.19)
twiceVA	−1.6 × 10^−5^ (≈0)	−7.7 × 10^−4^ (0.18)	−1.3 × 10^−3^ (0.31)

Higher genetic variation led to higher genetic changes for both traits. When considering twice the baseline genetic variance, the genetic changes in both vigour and sensitivity were enhanced by a factor ranging from 1.9 to 2.4 (excluding the drought stress‐free condition for sensitivity; Table 4). Being above 1.75, these values indicate that changing the initial genetic variance did more than theoretically expected for simple mass selection: This overeffect of the genetic variance on the response to selection can be explained by the role of fecundity selection, as shown above, and by the effect of the variance on the intensity of selective competition.

### 3.2 Feedback Effects of Genetic Evolution on Population Dynamics and Impacts of Drought Stress Regimes in the Unthinned Scenario

**FIGURE 3:**
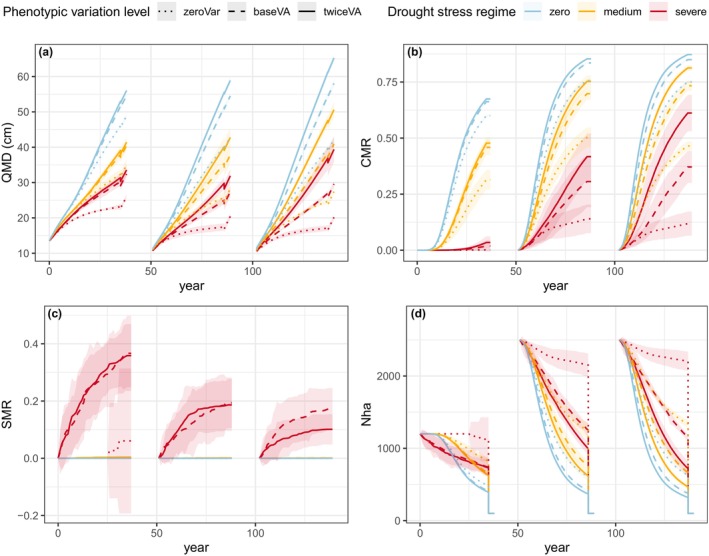
Dynamics of tree size and demography in the unthinned scenario across three successive rotations according to the level of phenotypic variation (line type) and drought stress regime (colours): (a) Quadratic mean diameter, QMD; (b) competition‐induced mortality rate, CMR; (c) drought stress‐induced mortality rate, SMR; and (d) number of trees per hectare, Nha. The shaded areas represent the 95% intervals over 10 replicates for each genetic setup. The prerecruitment period is intentionally omitted from the representation, as the model does not account for any demographic changes during this phase. Additionally, trees that have not yet reached recruitment age are not considered in the Nha.

With neither phenotypic variation for vigour and sensitivity nor drought stress, the quadratic mean diameter (QMD) progressively increased during stand development due to tree growth and selective competition‐induced mortality. QMD reached 49 cm at the end of the first rotation, which started with a low initial density analogous to plantation, then 43 cm at the end of the second and third rotation, which started with high initial density analogous to natural regeneration (Figure 3a). Competition‐induced mortality started at 19 years in the first rotation, and at 15 years (recruitment age) in the second and third rotations (Figure 3b). Without phenotypic variation, drought stress reduced growth (Figure 3a) and survival (Figure 3c). The medium and severe drought stress regimes reduced QMD at end of rotation by a factor of 1.39 and 1.88, respectively. Drought stress‐induced mortality occurred only under the severe drought stress regime, but at a low rate (< 0.08%). Moreover, by reducing growth and, to a lesser extent, survival, drought stress also reduced the intensity of competition and associated mortality, which began later during the first rotation (Figure 3b). Therefore, counterintuitively, higher drought stress was associated with lower overall mortality and increased density because the reduction in competition‐induced mortality outweighed drought stress‐induced mortality (Figure 3d). In other words, in our conditions, a more severe drought stress regime resulted in higher density of smaller trees.

With phenotypic variation, selection on vigour and sensitivity resulted in a substantial improvement in stand growth performance over generations, evident across all drought stress regimes (Figure 3a). Enhanced growth performance resulted in increased competition‐induced mortality (Figure 3b) and higher LAI (Appendix S3: Figure S1), hence higher drought stress level, thereby reinforcing selection on both vigour and sensitivity within a positive eco‐evolutionary feedback loop. With phenotypic variation, the overall impact of drought on growth was slightly mitigated by the genetic gain: medium and severe drought stress regimes reduced the QMD at the end of the rotation by a factor of 1.35 and 1.69, respectively, with baseline genetic variance, and by a factor of 1.35 and 1.67 with twice the baseline genetic variance (compared to 1.39 and 1.88 without phenotypic variation). Moreover, this mitigation of impacts on growth did not change over generations. Phenotypic variation introduced sensitive trees, leading to increased drought stress‐induced mortality under severe drought stress regimes. Conversely, selection against sensitivity resulted in a decrease in drought stress‐induced mortality across generations (Figure 3c): drought stress‐induced mortality rate (SMR) dropped from 0.37 at the end of the first rotation to 0.17 at the end of the third rotation with baseline genetic variance, and from 0.36 to 0.10 with twice the baseline genetic variance. This decline in drought stress‐induced mortality weakened the intensity of selection on sensitivity within a negative eco‐evolutionary feedback loop. However, this reduction in drought stress‐induced mortality was compensated by the resulting recovery of competition intensity, thereby reinforcing the soft selection process for both vigour and sensitivity (Figure 3b).

The following sections focus on the baseline genetic variance (baseVA) subjected to the severe drought stress regime and briefly describe the results obtained in the other cases. All cases are illustrated in Appendix S3.

### 3.3 Impact of Silvicultural Scenarios on Evolutionary Processes Under Drought Stress Regime

**FIGURE 4:**
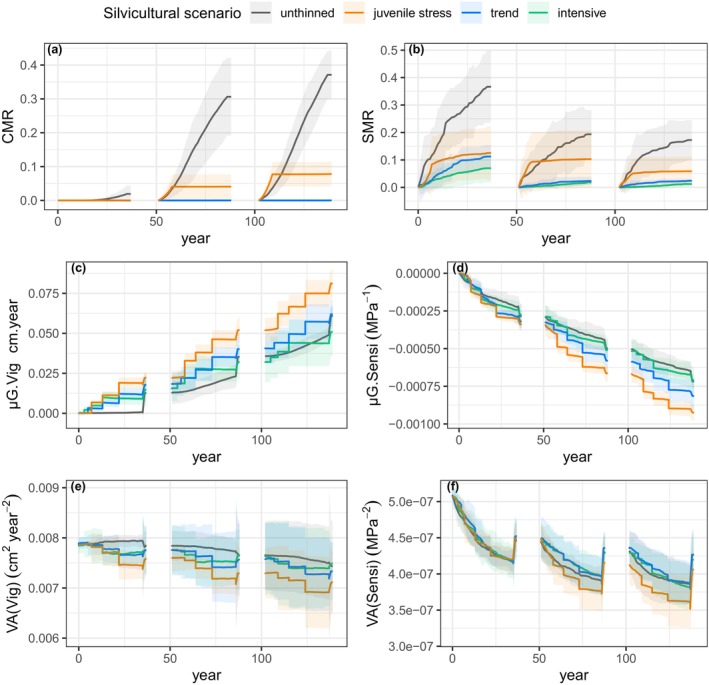
Dynamics of selective mortality and genetic changes across three successive rotations in four distinct silvicultural scenarios (colours): (a) Competition‐induced mortality rate, CMR; (b) drought stress‐induced mortality rate, SMR; (c) population genetic mean of vigour, μG.Vig; (d) population genetic mean of sensitivity, μG.Sensi; (e) additive genetic variance of vigour, VA(Vig); and (f) additive genetic variance of sensitivity, VA(Sensi). The thinning regimes for the different silvicultural scenarios are detailed in Table 2. This figure illustrates the baseline genetic variance (baseVA) under the severe drought stress regime; see Appendix S3: Figure S2 for the other drought stress regimes and levels of phenotypic variation. The shaded areas represent the 95% intervals over 10 replicates for each genetic setup. The prerecruitment period is intentionally omitted from the representation, as the model does not account for any demo‐genetic changes during this phase.

Under drought stress regimes, thinning interventions had antagonistic effects on genetic evolution. On the one hand, thinning reduced the intensity of natural selection on vigour and sensitivity. This reduction was achieved by lowering the drought stress level through the impacts on the dynamics of the LAI: The average drought stress level decreased by 1% in the juvenile stress scenario, 8% in the trend scenario and 13% in the intensive scenario (Appendix S3: Figure S1). Additionally, thinning replaced and, therefore, directly reduced the natural selective mortality induced by competition and drought stress. Thus, competition‐induced mortality did not occur throughout all three rotations in both the intensive and trend scenarios (Figure 4a). In the juvenile stress scenario, competition‐induced mortality did not occur during the first rotation, as thinning began before the onset of self‐thinning. However, in the early stage of the subsequent two rotations, competition‐induced mortality occurred, reaching a rate of 17% of that observed in the unthinned scenario in both rotations. Furthermore, in comparison to the unthinned scenario, the reduction in drought stress‐induced mortality was on average 88% in the intensive scenario, 81% in the trend scenario and 60% in the juvenile stress scenario, without distinction between rotations (Figure 4b). On the other hand, selective thinning favoured larger trees, that is, vigorous and/or less sensitive trees, resulting in pronounced shifts in the genetic mean of the two traits in the intensive, trend and juvenile stress scenarios (Figure 4c,d).

In this context, selective thinning appeared to be more effective than natural selection alone: Under the severe drought stress regime, the juvenile stress scenario amplified the genetic gain for vigour and against sensitivity after three generations by 33% and 29%, respectively, compared to the unthinned scenario (Figure 4c,d). The trend scenario increased the genetic gain for vigour and against sensitivity by 1% and 13%, respectively. By contrast, the intensive scenario reduced the genetic gain for vigour and against sensitivity by 17% and 1%, respectively. Meanwhile, the juvenile stress scenario led to a slightly higher reduction in genetic variances within each rotation compared to other silvicultural scenarios, but the genetic variances were partly restored at each regeneration phase (Figure 4e,f).

The ranking of the three scenarios with thinning interventions remained consistent across the different drought stress regimes and levels of genetic variation: The juvenile stress scenario showed the highest genetic gain, followed by the trend scenario and lastly, the intensive scenario (Appendix S3: Figure S2). By contrast, the position of the unthinned scenario was influenced by the drought stress regime and the level of genetic variation. Overall, more severe drought stress regimes lowered the ranking of the unthinned scenario in terms of genetic improvement. Specifically, in the absence of drought stress, the unthinned scenario exhibited the highest genetic changes among the silvicultural scenarios. Moreover, with twice the baseline genetic variance (twiceVA), the unthinned scenario tended to ascend in the ranking.

### 3.4 Overall Impact of Silvicultural Scenarios on Tree Size

**FIGURE 5:**
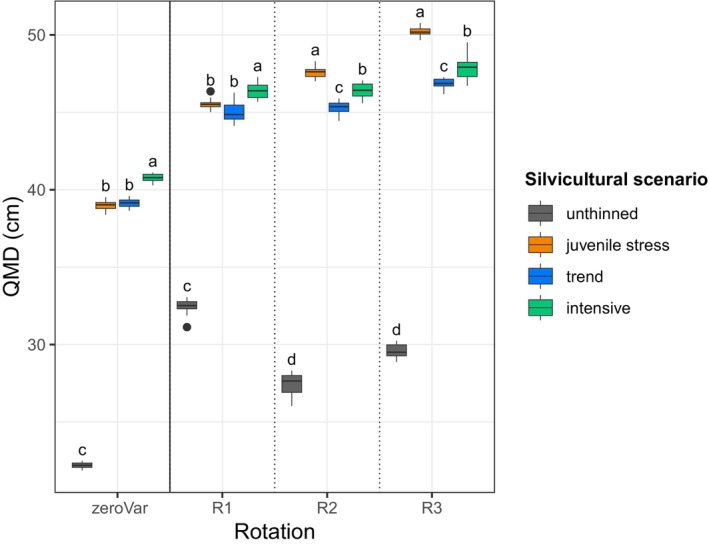
Quadratic mean diameter (QMD) at the end of three successive rotations (R1, R2 and R3) with phenotypic variation in four distinct silvicultural scenarios (colours). The thinning regimes for the different silvicultural scenarios are detailed in Table 2. The end‐of‐rotation quadratic mean diameter without phenotypic variation (zeroVar) is shown as an average across three rotations and is presented as a theoretical reference in the left panel. Tukey tests were performed independently for R1, R2, R3 and zeroVar, with letters indicating significance between silvicultural scenarios at the 0.05 threshold. In our simulations, the variability only arises from the stochastic components of the model. This figure illustrates the baseline genetic variance (baseVA) and the severe drought stress regime; see Appendix S3: Figure S3 for the other level of genetic variation (twiceVA) and other drought stress regimes. The boxplots show the distribution of values obtained from 10 replicates in each case.

Without phenotypic variation, the intensive scenario produced the largest end‐of‐rotation QMD, followed by the trend and juvenile stress scenarios, which were not significantly different; in contrast, the unthinned scenario displayed markedly lower values (Figure 5). Therefore, without phenotypic variation, the ranking of the silvicultural scenarios in terms of tree size was the opposite of their ranking in terms of drought stress and competition, reflecting the straightforward plastic response. By contrast, the introduction of phenotypic variation disrupted this ranking from one generation to the next due to differences in the genetic gain in vigour and sensitivity among silvicultural scenarios (Figure 5). Here, both competition and drought stress had a dual impact on tree size, by driving selection for genotypes with better growth (due to higher vigour, lower sensitivity or a combination of both) and simultaneously reducing the phenotypic expression of this genetic potential. In the first rotation, the juvenile stress scenario—characterised by the highest genetic gain—tended to outperform the trend scenario, although this difference was not statistically significant at the 0.05 threshold. Subsequently, in the following two generations, the juvenile stress scenario significantly exhibited the largest end‐of‐rotation QMD. Here, the genetic gain in increased vigour and reduced sensitivity were sufficient to offset the effects of competition. Meanwhile, the differences among the intensive, trend and unthinned scenarios tended to decrease.

The ranking of silvicultural scenarios in terms of end‐of‐rotation QMD was influenced by the drought stress regime and the level of genetic variation (Appendix S3: Figure S3). Across generations, the ranking of the end‐of‐rotation QMD generally tended to align with the ranking of genetic gain for vigour and against sensitivity. This convergence accelerated with a reduction in the severity of the drought stress regime and with twice the baseline genetic variance, that is, with increased genetic improvement, especially in terms of vigour.

## 4 Discussion

In this study, we employed a demo‐genetic modelling approach to gain novel insights into how thinning practices influence the eco‐evolutionary dynamics of naturally regenerated tree populations under drought stress regimes. Our simulation experiments covered drought stress levels observed across the entire European range of Douglas‐fir, including marginal stands, and considered plausible genetic variation for two fitness‐related traits: vigour and sensitivity to drought stress. We hypothesised that drought stress reduction strategies, such as intensive thinning, might reduce the intensity of selection on sensitivity, potentially hindering long‐term adaptation. Conversely, we expected that an evolution‐oriented forestry approach would promote genetic improvement over generations, leading to greater population resilience and adaptability to drought. Our findings support our hypotheses, revealing intricate interactions and feedback loops among natural adaptation processes, drought regimes, thinning practices and the resulting evolutionary trajectory of vigour and sensitivity within tree populations. We develop these points further in the following discussion.

### 4.1 Adaptive Potential of Unmanaged Tree Populations Under Drought

Our model highlights two natural selection processes co‐occurring in tree populations subjected to repeated droughts: Soft selection acting indirectly on both vigour and sensitivity through competition‐induced viability selection and fecundity selection on tree size, and hard selection acting on sensitivity through drought stress‐induced mortality. Consistently with prior findings on vigour alone using Luberon2 without disturbance (Godineau et al. 2023), the predicted evolutionary rates for both vigour and sensitivity in these simulations (from 0.08 to 0.41 over three generations) aligned with the observed rates of microevolution* documented for wild plant and animal populations, ranging from 0 to 0.65 (Bone and Farres 2001; Gingerich 2009; Bonnet et al. 2022). Furthermore, natural selection on vigour and sensitivity increased the mean diameter (QMD) by 13% per generation on average, which is comparable to the estimated genetic gain for growth classically observed in forest tree breeding programmes (e.g., 10%–25%, according to Jansson et al. 2017). Thus, the evolutionary rates and responses to selection predicted by the model are plausible. Nevertheless, they may be subject to positive and negative biases, as detailed below.

Several factors could contribute to an overestimation of evolutionary rates. First, in the simulations, the intensity of indirect natural selection on vigour and sensitivity is driven by their positive correlations with three fitness components: competing ability, ability to cope with drought stress and reproductive success. In reality, other processes not considered in the model could reduce these correlations and hamper the selection process. For instance, dominant trees are functionally more vulnerable to drought stress, experiencing greater growth reduction and higher mortality (Bennett et al. 2015). Furthermore, positive phenotypic correlations between vigour and sensitivity are often observed, indicating a trade‐off* between vigour and drought tolerance (Fririon et al. 2023). A positive genetic correlation between vigour and sensitivity induced by pleiotropy or by linkage disequilibrium in the initial population would reduce the correlation between each trait and the fitness components, that is, would reduce the selection gradients. Another factor that could contribute to overestimating evolutionary rates is the omission of mechanisms like adaptive phenotypic plasticity* or acclimation, which could mitigate drought stress impacts and reduce the intensity of selection against sensitivity (Paenke, Sendhoff, and Kawecki 2007; Nicotra et al. 2010; Cailleret et al. 2014). Moreover, our simulations did not consider gene flow from outside the population. If the source populations were genetically differentiated for the traits of interest, incoming gene flow would counteract local selection, resulting in overestimated evolutionary rates predicted by the model. But, in some cases of limited genetic variance, incoming gene flow could also favour the response to selection, meaning an underestimation of the evolutionary rates by the model. Given the size of the simulated forest (~10 ha), and the assumption that there are no highly differentiated gene pools nearby, we expect a limited amount of incoming gene flow with little impact on the predicted evolutionary rates. Conversely, a potential cause of underestimating evolutionary rates arises from our simulations overlooking that the ecological carrying capacity* of forests—modelled through a self‐thinning line in Luberon2—may decline as climatic or soil conditions degrade site quality. This is supported by empirical evidence from studies such as Forrester et al. (2021), Brunet‐Navarro et al. (2016) and Rodríguez de Prado et al. (2020), suggesting that under drought conditions, our model may underestimate selective mortality due to competition. Moreover, by assuming a stationary drought stress regime, we do not account for the effect of climate change. As climate conditions shift towards increased drought stress, we would expect greater selection pressure on sensitivity, alongside a reduced selection pressure on vigour. Lastly, the model's exclusion of prerecruitment events could have mixed effects on predictions of the evolutionary rates. On the one hand, it may overestimate both regeneration success and the number of seedlings recruited, particularly under drought conditions, potentially leading to an overestimation of selection pressure on vigour and sensitivity postrecruitment. On the other hand, by ignoring natural selection during the juvenile stage, the model may overlook a juvenile selective filter. The ultimate impact of juvenile selection on vigour and sensitivity is hardly predictable because drought sensitivity could vary with age (not considered in the model), while the intensity of competition, and the related fitness advantage provided by vigour, evolves during stand development. Despite these potential upward and downward biases in predicting selection responses, this study provides valuable insights into the dynamics of selection within and across generations.

As a dynamic process, natural selection varies throughout stand development, and DG‐ABM allows for such a dynamic approach to selection. Using Luberon2, we have identified six mechanisms associated with competition and drought stress that dynamically drive the selection processes within each rotation: (i) The correlation between the genetic value of growth‐related traits (vigour and sensitivity) and tree size—key fitness component driving survival and fecundity—gradually increases with age, thereby enhancing the indirect selection response of both traits; (ii) as the LAI increases during stand development, the drought stress level intensifies, strengthening the selection pressure on sensitivity while weakening the selection pressure on vigour; (iii) the competition‐induced mortality rate driving soft selection on both vigour and sensitivity decreases during stand development as population density decreases; (iv) the selection for high vigour and/or low sensitivity intensifies the levels of competition and drought stress in an eco‐evolutionary feedback loop; (v) the impacts of drought stress on growth and survival reduce competition and the corresponding selection pressure on both traits; and (vi) the drought stress‐induced mortailty rate (SMR) progressively decreases with the elimination of sensitive trees, leading to a reduction in the intensity of hard selection against sensitivity. Hence, the variation in genetic changes occurring within a single rotation is determined by the relative importance of each of these mechanisms, which is contingent upon the initial genetic composition and density of the stand, the environmental conditions (such as site index) and the disturbance regime.

For both traits and in all conditions, increasing the genetic variation of the initial population systematically increased the genetic changes in both vigour and sensitivity more than expected with the breeder's equation under the assumption of mass selection. This discrepancy can be attributed to different aspects accounted for in the demo‐genetic model but not in the breeder's equation, such as fecundity selection, dynamic changes in selection intensities or dynamic changes in the selection gradients of each trait. This highlights the suitability of a demo‐genetic approach in studying adaptive responses in nonstationary systems where the drivers of evolution exhibit their own dynamics through eco‐evolutionary feedback (Oddou‐Muratorio, Davi, and Lefèvre 2020; Lamarins et al. 2022).

### 4.2 Potential Mitigation of Drought Impacts Through Natural Selection

In our simulations, we found that drought impacts were influenced by the level of phenotypic variation. A consistent trend emerged where higher levels of phenotypic variation led to an overall mitigation of drought impacts on stand‐level growth, as evidenced by the lesser reduction in tree size observed with phenotypic variation compared to without it. However, despite clear and continued genetic improvement towards lower sensitivity with phenotypic variation, this mitigation effect did not improve across generations. We attribute this nuanced result to the combination of two processes mentioned earlier. First, recurrent droughts not only directly affect growth but also hinder selection of vigour across generations, resulting in a deficit in vigour compared to drought stress–free conditions. Second, the eco‐evolutionary feedback whereby drought stress levels increase with the selection of faster‐growing trees (vigorous and/or less sensitive) causes drought stress levels to increase over generations. In other words, the evolutionary response in sensitivity is essential to counteract the growing deficit in vigour and rising drought stress levels, ultimately resulting in the mitigation of the overall drought impacts on growth, as demonstrated in our simulations. Simultaneously, genetic improvement through natural selection against sensitivity directly reduced the drought stress‐induced mortality rate by an average of 0.1 per generation. Our findings globally align with a growing body of literature showcasing that, when selection is strong, demographic, ecological and evolutionary responses (rapid genetic adaptation) can all occur and interact within a relatively short timeframe, spanning just a few generations (Pelletier, Garant, and Hendry 2009; Hendry 2017; Govaert et al. 2019). Furthermore, these eco‐evolutionary mechanisms underlie the process of evolutionary rescue*, enabling a population to persist through selective pressures in response to rapidly changing environmental conditions, such as climate change, that would otherwise have led to its extinction (Gomulkiewicz and Holt 1995; Carlson, Cunningham, and Westley 2014; Gloy, Herzschuh, and Kruse 2023).

### 4.3 Silviculture as a Lever for Enhancing Genetic Adaptation While Preserving Long‐Term Evolvability: Towards Evolution‐Oriented Forestry

Silviculture plays a dual role in evolutionary dynamics: It partially replaces natural evolutionary processes, as already demonstrated by Godineau et al. (2023), and simultaneously serves as a driver of anthropogenic selection, as illustrated in this study. Aside from the first thinning of the trend and intensive scenarios, which was random, thinning selectively targeted smaller trees (thinning from below). Thus, thinning acted in the same direction as natural selection on growth‐related traits through self‐thinning and fecundity selection. In these simulations, the genetic improvement of the population against sensitivity was more efficient with anthropogenic selection than solely with natural selection, especially when the impacts of drought stress reduced the intensity of selective competition that drives natural selection. The increase in genetic improvement due to management, which we qualify as a form of ‘breeding by silviculture’, was not associated with significant erosion of the standing genetic variance. This result is consistent with the meta‐analysis conducted by Hendry, Farrugia, and Kinnison (2008) in naturally evolving animal populations with some management, like forests, showing that evolutionary rates tend to be higher in human‐influenced environments compared to natural contexts.

In reality, foresters are likely to adopt a range of strategies to determine which trees to harvest or retain. This can result in thinning from below, systematic thinning, thinning from above or any combination of those depending on local objectives, population health and professional judgement of the forester. Each approach may have multiple, both positive and negative, effects on the evolutionary trajectory of the populations. Specifically, thinning from above, such as high grading, is likely to actively reduce the vigour of the population and increase its sensitivity. However, we do not anticipate that it might have a different impact on genetic variance compared to thinning from below.

Thinning has widely demonstrated its effectiveness in reducing drought stress, thereby improving the growth and survival of the remaining trees (Sohn, Saha, and Bauhus 2016; Wang et al. 2019; Schmitt et al. 2020; Gavinet et al. 2020; Moreau et al. 2022). Luberon2 effectively demonstrated its ability to replicate these functional responses. However, in line with our working hypothesis, the simulations demonstrate that intensive thinning regimes—characterised by heightened intensity, frequency and juvenile random thinning—globally reduce the selection differential* in sensitivity. Moreover, silvicultural strategies aimed at promoting genetic evolution can result in higher stand performance in terms of growth compared to strategies based on drought stress reduction only. The genetic evolution in both vigour and sensitivity has the potential to counteract the impact of competition and drought stress on growth, which occurred within just two generations in our simulations using the juvenile stress scenario under severe drought stress regime. Quantitatively, the potential benefits of such strategies promoting evolutionary changes depend on the level of within‐stand genetic variation in vigour and sensitivity.

The juvenile stress scenario, designed for even‐aged forest systems, is a first attempt to establish a balance between drought stress mitigation and evolutionary benefits, aiming to reconcile short‐ and long‐term forest objectives. This strategy involves promoting both natural and anthropogenic selection by avoiding early and nonselective thinnings, which can be combined with density reduction to mitigate drought stress in later stages. The juvenile stress strategy, by increasing exposure to drought stress, may also promote acclimation as another process leading to resilience not modelled here (Montwé, Spiecker, and Hamann 2015). This evolution‐oriented strategy acknowledges and drives microevolutionary processes (Lefèvre et al. 2014). However, the effectiveness of such an approach is contingent upon the local genetic diversity, as Schueler et al. (2021) demonstrated substantial variations in the heritability and evolvability of drought‐related traits among tree populations and species. Additionally, practical implementation should consider juvenile–mature genetic correlations that could alter adaptability (Gwaze et al. 2000), which are not incorporated in the current version of the model. Real‐world management also faces multirisk management issues (Jactel et al. 2009; Duncker et al. 2012; Seidl et al. 2017), including potential trade‐offs between tolerance to a plethora of stressors and growth performance (Kleinhentz, Jactel, and Raffin 1998; Bansal, Harrington, and St. Clair 2016). Furthermore, implementing a juvenile stress management strategy could introduce financial risks to the forester. These risks can arise from various factors, including an overdensity of the stand, which would require additional specific interventions for the progressive establishment of a robust stand structure, a potential extension of rotations or a potential reduction of the capacity to select the trees to keep on other criteria, for example, wood quality. Ultimately, while accelerating evolution is particularly desirable in the context of climate change, timing is critical, as silvicultural practices based on phenotype may use selection criteria misaligned with future adaptive needs. For instance, in emerging drought stress environments, selecting large trees may inadvertently focus selection primarily on vigour, or even favour drought‐sensitive trees when vigour and drought tolerance are negatively correlated (Fririon et al. 2023).

## 5 Conclusion

This study enhances our understanding of eco‐evolutionary feedback loops in populations subject to intraspecific competition and recurrent disturbances like droughts. Using the Luberon2 model, it simulates the dynamic impacts of drought on tree growth, reproduction and survival, accounting for feedback between drought stress and stand dynamics in a naturally regenerated forest. This approach offers quantitative insights into natural selection processes and evolutionary rates for two fitness‐related traits, vigour and sensitivity to drought stress, while also evaluating how natural selection may mitigate the impacts of drought. Our results may have three main applications for management. Firstly, the predicted evolutionary rates in terms of genetic gain or loss in vigour and sensitivity can be compared to locally expected disturbance regimes for the assessment of forests' vulnerability from a dynamic perspective. Secondly, the insights from the Luberon2 simulations could be used to design and test adaptive silvicultural scenarios, with long‐time monitoring of real forest stands over time to track how eco‐evolutionary processes unfold and guide adaptive management strategies. Thirdly, the predicted impact of increasing the genetic variance can be used to design genetic enrichment, assisted gene flow management (Aitken and Whitlock 2013) or species introduction strategies. Luberon2 offers the potential for extension to address multirisk issues by integrating additional abiotic or biotic disturbance regimes affecting growth, reproduction or survival. It can also be adapted to include nonstationary stress regimes reflecting climate change impacts. Moreover, this study highlights how the demo‐genetic agent‐based modelling framework can be used to incorporate eco‐evolutionary considerations into adaptive management planning. In the forestry case, we explored the dual role of management in influencing both the direct impacts of disturbances and the adaptation process, fostering a broader reflection on evolution‐oriented forestry practices. Obviously, plantation forests where the planting stock is chosen after each rotation are not concerned by the long‐term genetic consequences revealed in this study across multiple generations, but they are concerned by the short‐term impacts of eco‐evolutionary feedback revealed over one rotation. Similar modelling approaches could be developed for other agroecological systems, whether for production, conservation or any combination of these objectives.

## Conflicts of Interest

The authors declare no conflicts of interest.

## Supporting information


**Appendix S1.** Glossaries of genetics and forestry terms.

**Appendix S2.** Calibration of three drought stress regime generators.

**Appendix S3.** Supplementary figures.

**Appendix S4.** Correcting the impacts of phenotypic variation in vigour on growth prediction by a demo‐genetic coupled model.

## Data Availability

The dataset associated with this publication is archived and publicly available in the French Recherche Data Gouv multidisciplinary repository under DOI https://doi.org/10.57745/SY7NYH. The archive includes all necessary elements to reproduce the research: (i) a stand‐alone version of the Luberon2 model; (ii) the input and output files of the simulations presented in this work; and (iii) the R scripts used to analyse the simulation outputs and reproduce the figures presented in the publication.

## Endnotes

^1^
This interdisciplinary study lies at the intersection of evolutionary biology and forestry sciences. To facilitate reading for nonspecialists in each of these fields, we have included two glossaries (Appendix S1), each dedicated to the terminology of its respective discipline. When they first occur, terms defined in one of these glossaries are marked with an asterisk (*).
