# Supplementary material for: Can Thinning Foster Forest Genetic Adaptation to Drought? A Demo‐Genetic Modelling Approach With Disturbance Regimes
Source: Evol Appl. 2024 Dec 9;17(12):e70051. doi: 10.1111/eva.70051 (PMC11627118; doi:10.1111/eva.70051)
Supplement: Supplementary file 1 — Appendix S1. Glossaries of genetics and forestry terms. [file EVA-17-e70051-s003.docx]

Victor Fririon, Hendrik Davi, Sylvie Oddou-Muratorio, Gauthier Ligot, François Lefèvre

**Can Thinning Foster Forest Genetic Adaptation to Drought? A Demo-Genetic Modelling Approach with Disturbance Regimes**

# Appendix S1: Glossaries of Genetics and Forestry Terms

To report on this interdisciplinary work, we could not avoid using some specific terms of evolutionary biology and forestry sciences. In two short glossaries, we provide the definition of key terms of each discipline that are used but not explicitly defined in the publication. For other definitions, many good manuals are available, among which we can cite Lynch & Walsh (1998), in genetics, and Nyland et al. (2016), in silviculture and forestry.

## Glossary of Genetic and Evolutionary Terms

| **Term** | **Definition** |
| --- | --- |
| *Adaptation* | Adaptation is a particularly polysemic term. In its strict sense, it refers to the dynamic evolutionary process by which a population becomes better suited to its environment through genetic changes that enhance traits associated with survival and reproduction, thereby increasing fitness. When selection is strong, rapid adaptation can occur in just a few generations (Govaert et al., 2019; Bonnet et al., 2022; Gloy et al., 2023).  This evolutionary sense of adaptation, defined at population level, differs from acclimation, which involves non-heritable adjustments that organisms make during their lifetime (Kleine et al., 2021). Both contribute to adaptation in a broad sense (Aubin et al., 2016). |
| *Additive Genetic Variance* | The portion of genetic variance in a population that is attributed to the additive effects of individual alleles. |
| *Allele* | A specific version of a gene. Each gene can have multiple alleles (a diallelic gene has only two possible alleles) and, in diploid organisms (like most tree species), each individual carries two alleles of each gene that may be identical or different. The allelic variation is responsible for the genetic variation in traits seen among individuals within and across populations. |
| *Breeder's Equation* | A fundamental equation that predicts the genetic response of a population to mass selection and random mating (Lynch & Walsh, 1998; the equation is first attributed to Lush, 1937). It is expressed as *R = h².S*, where *R* is the response to selection, *h²* is the narrow sense heritability, and *S* is the selection differential. Alternatively, it can be expressed as *R = i.h.σA*, where *i* is the selection intensity, *h* is the square root of the narrow-sense heritability, and *σA* is the square root of the additive genetic variance. |
| *Demo-Genetic Agent-Based Models (DG-ABM)* | Individual-based (meta) population dynamics models with heritable trait variation and phenotype-dependent interactions between individuals (Lamarins et al., 2022). These models integrate both demographic and genetic components, allowing for the examination of how ecological and evolutionary processes interact when they occur over similar time scales. DG-ABMs explicitly model the feedback loop between ecological and evolutionary processes. |
| *Eco-evolution* | Refers to the interplay between ecological and evolutionary processes over time (Pelletier et al., 2009). This concept encompasses how ecological interactions, such as competition, influence evolutionary adaptations and vice versa. Eco-evolutionary feedback occurs when ecological dynamics drive evolutionary changes in populations, which in turn affect ecological processes. |
| *Evolution-Oriented Forestry* | Forest management practices accounting for, and taking benefit of natural selection processes to enhance genetic adaptation and evolutionary processes of tree populations (Lefèvre et al., 2014). It can be considered as an "environmental stewardship" approach of forestry (Mathevet et al., 2018), fostering local adaptation to specific conditions and contributing to adaptive management amidst ecological uncertainty (Marmorek et al., 2006; Garmestani & Allen, 2015). |
| *Evolutionary Rescue* | A demo-genetic process through which a population—that would have gone extinct in the absence of genetic evolution—persists a sudden environmental change due to natural selection acting on heritable variation (Gomulkiewicz & Holt, 1995; Carlson et al., 2014). |
| *Evolutionary Trade-Off* | Refers to the balance between traits where enhancing the fitness associated with one trait comes at the cost of reduced fitness in another trait. This concept highlights the constraints on evolutionary progress, where gains in one area may be offset by losses in another (e.g., Kleinhentz et al., 1998; Bansal et al., 2016). |
| *Fitness* | Fitness may have slightly different definitions, as reviewed by Orr (2009). Most generally, it is a quantitative measure of an individual's reproductive success, reflecting its ability to pass on its alleles to the next generation. When fitness is referred to at the population level, it represents the overall ability of the population to survive and thrive in a given environment, reflecting the cumulative contributions of individuals to both reproduction and survival. |
| *Gene Flow* | The transfer of genetic variation from one population to another, by any available means, for example through sexual reproduction (Savolainen et al., 2007). |
| *Genetic Architecture* | The underlying genetic determinism of a trait, including the number, location, individual effects of genes and alleles, and their possible interactions that contribute to genetic variation. |
| *Genetic Correlation* | The proportion of variance shared between two traits that is attributable to genetic factors. This correlation arises from genetic causes such as linkage disequilibrium, where alleles at different loci are not independently distributed among individuals, or/and pleiotropy, where a single gene influences multiple traits (e.g., Kleinhentz et al., 1998; Gwaze et al., 2000). |
| *Genetic Drift* | The change in allele frequencies within a small population due to random sampling of alleles from one generation to the next. |
| *Genetic Gain* | This term emerged in the 1940’s in the breeders’ community (Rutkoski, 2019). It refers to the genetic improvement in performance of a breeding population per generation. Here, we extend its use to naturally regenerated forests, where the forester acts as the breeder by promoting favourable genotypes throughout stand development and selecting the best trees for breeding from the remaining population. |
| *Genotypic Value* | The overall genetic value of an individual for a specific trait, considering the cumulative effect of all alleles at all loci related to that trait. |
| *Hard Selection* | A form of natural selection in which an individual's absolute fitness depends solely on its own trait value, regardless of the phenotypic composition of the population (e.g., drought tolerance) (Bell et al., 2021). |
| *Heritability (h²)* | The proportion of total phenotypic variation in a population that is due to genetic differences among individuals. It ranges from 0 to 1. In a broad sense, it includes all genetic contributions to phenotypic variation, such as additive effects and gene interactions. In a narrow sense, it focuses specifically on the additive genetic variance, which is directly related to inheritance. |
| *Indirect Selection* | Occurs when a trait evolves not because it directly influences fitness, but because it is correlated with another trait that is under direct selection. |
| *Linkage disequilibrium* | Refers to the non-random association of alleles at different loci in a population. In other words, the presence of a specific allele at one locus is correlated with the presence of an allele at another locus. It can be caused by physical proximity of loci on the chromosome, natural selection, gene flow, mutations, or/and genetic drift. |
| *Local Adaptation* | An evolutionary process in which a population evolves to become better suited to its local environment compared to other populations of the same species living in different environments (e.g., Lytle, 2001; Tonnabel et al., 2012; Malíková et al., 2016; Bansal et al., 2016). Local adaptation occurs when different populations experience distinct selective pressures, and its extent depends on the balance between gene flow and selection pressures (Savolainen et al., 2007). |
| *Microevolution* | Refers to changes in allele frequencies over small time-scale within a population (e.g., Bone & Farres, 2001; Gingerich, 2009; Bonnet et al., 2022). These changes result from processes like natural selection, genetic drift, mutation, and gene flow, and they lead to evolutionary changes within a species. |
| *Phenotypic Plasticity* | As a general definition, plasticity is defined at the level of a trait of an organism as its capacity to change in response to environmental conditions. Phenotypic plasticity can be adaptive or maladaptive, it can also vary genetically, with various ecological and evolutionary consequences (Nussey et al., 2007; Chevin et al., 2013). |
| *Phenotypic Value* | The observable expression of a trait in an individual, resulting from the combination of genetic and environmental factors. It is the measurable outcome of a specific trait. |
| *Phenotypic Variance* | A measure of the phenotypic variation in a quantitative trait among individuals within a population, encompassing both genetic and environmental factors. |
| *Quantitative Genetics* | The branch of genetics that studies the inheritance of traits that are influenced by multiple genes. It focuses on understanding how genetic and environmental factors contribute to the variation in these traits within a population. |
| *Quantitative Trait* | A phenotypic characteristic that is influenced by multiple genes, with different individual effects and possible interactions, and generally exhibits continuous variation, like growth rate, wood quality or tolerance to various stresses. |
| *Quantitative Trait Locus (QTL)* | One of the multiple loci that contribute to the variation of a quantitative trait. |
| *Response to Selection (R)* | The change in the average value of a trait in a population over generations due to selection. It measures how much a trait value shifts as a result of applying selection pressure. |
| *Selection Differential (S)* | The difference between the average trait value of selected individuals and the average trait value of the entire population before selection. It is related to the intensity of selection applied to a population. |
| *Selection Gradient* | The response of relative fitness to the variation of a trait, quantifying how changes in the trait's value influence an individual's reproductive success or survival. |
| *Selection Intensity (i)* | It is a standardised measure of the strength of selection on a trait defined as the selection differential divided by the phenotypic standard deviation. It enables comparison of selection strength across different traits or populations by normalising the selection differential relative to the trait's variability. |
| *Sensitivity (to drought stress)* | A quantitative trait that determines how much the growth of a tree, which ultimately also contributes to survival and reproduction, is affected by drought stress. For each individual tree, it is proxied by the slope of the relationship between radial growth and drought stress level (Fririon et al., 2023). |
| *Soft Selection* | A form of natural selection where an individual's absolute fitness is determined by its trait value relative to the traits of other members of the same species with which it interacts (e.g., competition-related traits) (Bell et al., 2021). |
| *Vigour* | A quantitative trait that determines the growth potential of a tree under optimal, non-stress conditions. For each individual tree, it is proxied by the deviation from the average stand growth under such conditions (Fririon et al., 2023). |

## Glossary of Silviculture and Forestry Terms

| **Term** | **Definition** |
| --- | --- |
| *Basal Area (BA)* | The cross-sectional area of a tree trunk measured at 1.30 metres above the ground. When expressed per hectare, it is the sum of the basal areas of all trees on that hectare, used to measure forest density. |
| *Diameter at breast height (DBH)* | The diameter of a tree measured at breast height, which is 1.3 metres above the ground. It is a standard measurement in forestry. |
| *Carrying Capacity* | The maximum number of living individuals of a species that the environment can support, given the available resources such as water and nutrients (Rodríguez de Prado et al., 2020).  In forest, the carrying capacity can be modelled by a self-thinning line defining the maximum tree density for a given average tree size (e.g., Ligot et al., 2023). |
| *Dendrometric Variables* | Variables related to the measurement of trees, including height, diameter, volume and basal area. |
| *Even-Aged Stand* | A type of forest stands where the trees are of approximately the same age or within a narrow age range. This age uniformity is typically the result of a single regeneration event (including plantations). They have a simplified structure compared to uneven-aged forests. |
| *Final Harvest* | The removal of all trees in an even-aged stand. This intervention typically occurs at the end of a rotation period, once the stand has reached a specified maturity or target condition. The final harvest aims to clear the stand of all remaining trees to prepare the site for a new regeneration cycle, either through natural processes or planting. |
| *Forest Management* | The process of planning and implementing interventions to maintain and improve the health, productivity, and sustainability of forest ecosystems. This includes planning, silviculture, harvesting, regeneration, monitoring, and protection to balance ecological, economic, and social objectives. |
| *Leaf Area Index (LAI)* | The ratio of leaf area to ground area in a forest stand, classically used to quantify the amount of leaf material and its effect on factors like light interception and drought stress (e.g., Smith, 1993; Bréda et al., 1995). |
| *Pre-Commercial Thinning* | A forest management practice of removing a fraction of the living trees. It is carried out in dense and relatively young stands before they reach a marketable size. This practice is generally used to improve the growing conditions of the remaining trees. |
| *Quadratic Mean Diameter (QMD)* | The geometric or quadratic mean of tree diameter, a standard metric for characterising stand structure and development stage (Curtis & Marshall, 2000); is calculated for the entire stand or by species in mixed forests. |
| *Recruitment* | The process by which new seedlings or young trees become established and are greater than a threshold size. It is usually the same diameter threshold as the one used during forest survey (only the trees with diameter greater than this threshold are measured). Recruitment represents the addition of new individuals (the recruits) to the population, contributing to forest regeneration and succession.  The recruitment age refers to the age at which these young trees reach the threshold size and are included in the population count. |
| *Regeneration* | The process by which a forest is rejuvenated, either naturally relying on natural seed dispersal and germination or natural sprouting (e.g., Petit & Claessens, 2013), or artificially through human interventions, such as planting seedlings or sowing seeds. |
| *Rotation* | The period of time between the regeneration of a forest stand and its final harvest (in even-aged stands). This cycle encompasses all stages of forest development, from establishment through growth and maturation, until the stand is harvested and replanted or naturally regenerated. |
| *Seed Tree* | A mature tree left standing to provide seeds for the regeneration of a harvested stand. |
| *Seeding (or Regeneration) Cut* | A silvicultural practice where a portion of mature trees is left standing to provide seeds for natural regeneration before final harvest. |
| *Self-Thinning* | A natural process where, as trees grow and compete for resources such as light, water, and nutrients, some trees, mostly the small and suppressed ones, die off (Brunet-Navarro et al., 2016; Forrester et al., 2021). This process reduces the density of trees, allowing the remaining ones to continue growing and improving overall stand health and productivity. |
| *Silvicultural Scenario* | A planned sequence of forest management interventions designed to achieve specific objectives in a forest stand. These scenarios typically involve various interventions such as thinning, harvesting, and regeneration techniques tailored to meet goals like sustaining the growth of particular trees, improving forest health, or enhancing biodiversity. |
| *Silviculture* | The practice of managing forest stands to drive their establishment, growth, composition, and quality (Nyland et al., 2016; Achim et al., 2022). This involves interventions such as thinning, harvesting, and seeding cuts to achieve specific objectives, including timber production, ecological balance, or habitat enhancement. |
| *Site Index* | A measure used in forestry to evaluate the productivity of a site based on the height of the dominant trees at a specific age (usually 25 or 50 years). It provides an indication of the potential growth capacity of a forest site and helps in assessing its suitability for timber production. |
| *Thinning* | The partial cut of a stand to reduce competition, improve growth rates, and enhance forest health. Thinning can be tailored to specific objectives and may involve removing larger, dominant trees (thinning from above), smaller, suppressed trees (thinning from below), or trees independent of size (e.g., systematic thinning).  Thinning from below is commonly practised in production-oriented conifer stands, including Douglas-fir, in an effort to concentrate resources on the best-growing trees and enhance the production of high-quality timber (Mäkinen & Isomäki, 2004; Perin et al., 2016). |

## References

Achim, A., G. Moreau, N. C. Coops, J. N. Axelson, J. Barrette, S. Bédard, K. E. Byrne, et al. 2022. ‘The Changing Culture of Silviculture’. *Forestry: An International Journal of Forest Research* 95 (2): 143–52. https://doi.org/10.1093/forestry/cpab047.

Aubin, I., A.D. Munson, F. Cardou, P.J. Burton, N. Isabel, J.H. Pedlar, A. Paquette, et al. 2016. ‘Traits to Stay, Traits to Move: A Review of Functional Traits to Assess Sensitivity and Adaptive Capacity of Temperate and Boreal Trees to Climate Change’. *Environmental Reviews* 24 (2): 164–86. https://doi.org/10.1139/er-2015-0072.

Bansal, S., C. A. Harrington, and J. B. St. Clair. 2016. ‘Tolerance to Multiple Climate Stressors: A Case Study of Douglas-Fir Drought and Cold Hardiness’. *Ecology and Evolution* 6 (7): 2074–83. https://doi.org/10.1002/ece3.2007.

Bell, D. A., R. P. Kovach, Z. L. Robinson, A. R. Whiteley, and T. E. Reed. 2021. ‘The Ecological Causes and Consequences of Hard and Soft Selection’. *Ecology Letters* 24 (7): 1505–21. https://doi.org/10.1111/ele.13754.

Bone, E., and A. Farres. 2001. ‘Trends and Rates of Microevolution in Plants’. *Genetica* 112 (1): 165–82. https://doi.org/10.1023/A:1013378014069.

Bonnet, T., M. B. Morrissey, P. de Villemereuil, S. C. Alberts, P. Arcese, L. D. Bailey, S. Boutin, et al. 2022. ‘Genetic Variance in Fitness Indicates Rapid Contemporary Adaptive Evolution in Wild Animals’. *Science* 376 (6596): 1012–16. https://doi.org/10.1126/science.abk0853.

Bréda, N., A. Granier, and G. Aussenac. 1995. ‘Effects of Thinning on Soil and Tree Water Relations, Transpiration and Growth in an Oak Forest (Quercus Petraea (Matt.) Liebl.)’. *Tree Physiology* 15 (5): 295–306. https://doi.org/10.1093/treephys/15.5.295.

Brunet-Navarro, P., F. J. Sterck, J. Vayreda, J. Martinez-Vilalta, and G. M. J. Mohren. 2016. ‘Self-Thinning in Four Pine Species: An Evaluation of Potential Climate Impacts’. *Annals of Forest Science* 73 (4): 1025–34. https://doi.org/10.1007/s13595-016-0585-y.

Carlson, S. M., C. J. Cunningham, and P. A. H. Westley. 2014. ‘Evolutionary Rescue in a Changing World’. *Trends in Ecology & Evolution* 29 (9): 521–30. https://doi.org/10.1016/j.tree.2014.06.005.

Chevin, L.-M., S. Collins, and F. Lefèvre. 2013. ‘Phenotypic Plasticity and Evolutionary Demographic Responses to Climate Change: Taking Theory out to the Field’. *Functional Ecology* 27 (4): 967–79. https://doi.org/10.1111/j.1365-2435.2012.02043.x.

Curtis, R. O., and D. D. Marshall. 2000. ‘Technical Note: Why Quadratic Mean Diameter?’ *Western Journal of Applied Forestry* 15 (3): 137–39. https://doi.org/10.1093/wjaf/15.3.137.

Forrester, D. I., T. G. Baker, S. R. Elms, M. L. Hobi, S. Ouyang, J. C. Wiedemann, W. Xiang, J. Zell, and M. Pulkkinen. 2021. ‘Self-Thinning Tree Mortality Models That Account for Vertical Stand Structure, Species Mixing and Climate’. *Forest Ecology and Management* 487 (May):118936. https://doi.org/10.1016/j.foreco.2021.118936.

Fririon, V., H. Davi, S. Oddou-Muratorio, F. Lebourgeois, and F. Lefèvre. 2023. ‘Within and between Population Phenotypic Variation in Growth Vigor and Sensitivity to Drought Stress in Five Temperate Tree Species’. *Forest Ecology and Management* 531 (March):120754. https://doi.org/10.1016/j.foreco.2022.120754.

Garmestani, A., and C. Allen. 2015. ‘Adaptive Management of Social-Ecological Systems: The Path Forward’. *Adaptive Management of Social-Ecological Systems*, April, 255–62. https://doi.org/10.1007/978-94-017-9682-8_14.

Gingerich, P. D. 2009. ‘Rates of Evolution’. *Annual Review of Ecology, Evolution, and Systematics* 40 (1): 657–75. https://doi.org/10.1146/annurev.ecolsys.39.110707.173457.

Gloy, J., U. Herzschuh, and S. Kruse. 2023. ‘Evolutionary Adaptation of Trees and Modelled Future Larch Forest Extent in Siberia’. *Ecological Modelling* 478 (April):110278. https://doi.org/10.1016/j.ecolmodel.2023.110278.

Gomulkiewicz, R., and R. D. Holt. 1995. ‘When Does Evolution by Natural Selection Prevent Extinction?’ *Evolution* 49 (1): 201–207.

Govaert, L., E. A. Fronhofer, S. Lion, C. Eizaguirre, D. Bonte, M. Egas, A. P. Hendry, et al. 2019. ‘Eco-Evolutionary Feedbacks—Theoretical Models and Perspectives’. *Functional Ecology* 33 (1): 13–30. https://doi.org/10.1111/1365-2435.13241.

Gwaze, D. P., F. E. Bridgwater, T. D. Byram, J. A. Woolliams, and C. G. Williams. 2000. ‘Predicting Age-Age Genetic Correlations in Tree-Breeding Programs: A Case Study of Pinus Taeda L.’ *Theoretical and Applied Genetics* 100 (2): 199–206. https://doi.org/10.1007/s001220050027.

Kleine, T., T. Nägele, H. E. Neuhaus, C. Schmitz-Linneweber, Alisdair R. Fernie, P. Geigenberger, B. Grimm, et al. 2021. ‘Acclimation in Plants – the Green Hub Consortium’. *The Plant Journal* 106 (1): 23–40. https://doi.org/10.1111/tpj.15144.

Kleinhentz, M., H. Jactel, and A. Raffin. 1998. ‘Genetic Parameters and Gain Expected from Direct Selection for Resistance to Dioryctria Sylvestrella Ratz. (Lepidoptera: Pyralidae) in Pinus Pinaster Ait., Using a Full Diallel Mating Design’. *International Journal of Forest Genetics*. https://agris.fao.org/agris-search/search.do?recordID=SK2000000171.

Lamarins, A., V. Fririon, D. Folio, C. Vernier, L. Daupagne, J. Labonne, M. Buoro, F. Lefèvre, C. Piou, and S. Oddou-Muratorio. 2022. ‘Importance of Interindividual Interactions in Eco-Evolutionary Population Dynamics: The Rise of Demo-Genetic Agent-Based Models’. *Evolutionary Applications* 15 (12): 1988–2001. https://doi.org/10.1111/eva.13508.

Lefèvre, F., T. Boivin, A. Bontemps, F. Courbet, H. Davi, M. Durand-Gillmann, B. Fady, et al. 2014. ‘Considering Evolutionary Processes in Adaptive Forestry’. *Annals of Forest Science* 71 (7): 723–39. https://doi.org/10.1007/s13595-013-0272-1.

Ligot, G., T. Gheysen, J. Perin, R. Candaele, F. de Coligny, A. Licoppe, and P. Lejeune. 2023. ‘From the simulation of forest plantation dynamics to the quantification of bark-stripping damage by ungulates’. *European Journal of Forest Research*, April. https://doi.org/10.1007/s10342-023-01565-w.

Lush, J. L. 1937. *Animal Breeding Plans*. Ames, Ia.: Collegiate Press, Inc.

Lynch, M., and B. Walsh. 1998. *Genetics and Analysis of Quantitative Traits*. 1st edition. Sunderland, Mass: Sinauer Associates is an imprint of Oxford University Press.

Lytle, D. A. 2001. ‘Disturbance Regimes and Life-History Evolution’. *The American Naturalist* 157 (5): 525–36. https://doi.org/10.1086/319930.

Mäkinen, H., and A. Isomäki. 2004. ‘Thinning Intensity and Growth of Scots Pine Stands in Finland’. *Forest Ecology and Management* 201 (2): 311–25. https://doi.org/10.1016/j.foreco.2004.07.016.

Malíková, L., V. Latzel, P. Šmilauer, and J. Klimešová. 2016. ‘Local Adaptation of Annual Weed Populations to Habitats Differing in Disturbance Regime’. *Evolutionary Ecology* 30 (5): 861–76. https://doi.org/10.1007/s10682-016-9845-4.

Marmorek, D. R., D. C. E. Robinson, C. Murray, and L. Greig. 2006. ‘Enabling Adaptive Forest Management’. https://doi.org/10.13140/2.1.2301.5367.

Mathevet, R., F. Bousquet, and C. M. Raymond. 2018. ‘The Concept of Stewardship in Sustainability Science and Conservation Biology’. *Biological Conservation* 217 (January):363–70. https://doi.org/10.1016/j.biocon.2017.10.015.

Nussey, D. H., A. J. Wilson, and J. E. Brommer. 2007. ‘The Evolutionary Ecology of Individual Phenotypic Plasticity in Wild Populations’. *Journal of Evolutionary Biology* 20 (3): 831–44. https://doi.org/10.1111/j.1420-9101.2007.01300.x.

Nyland, R. D., L. S. Kenefic, K. K. Bohn, and S. L. Stout. 2016. *Silviculture: Concepts and Applications, Third Edition*. 3rd edition. Long Grove, Illinois: Waveland Press, Inc.

Orr, H. A. 2009. ‘Fitness and Its Role in Evolutionary Genetics’. *Nature Reviews Genetics* 10 (8): 531–39. https://doi.org/10.1038/nrg2603.letier, F., D. Garant, and A. P. Hendry. 2009. ‘Eco-Evolutionary Dynamics’. *Philosophical Transactions of the Royal Society B: Biological Sciences* 364 (1523): 1483–89. https://doi.org/10.1098/rstb.2009.0027.

Perin, J., J. Hebert, P. Lejeune, and H. Claessens. 2016. ‘De nouvelles normes sylvicoles pour les futaies pures équiennes d’épicéa et de douglas en appui à la gestion de la forêt publique en Wallonie’. Forêt Wallonne asbl. https://orbi.uliege.be/handle/2268/234913.

Petit, S., and H. Claessens. 2013. ‘La régénération naturelle des douglasaies a le vent en poupe. Le point sur les itinéraires techniques existants’, 13.

Rodríguez de Prado, D., R. San Martín, F. Bravo, and C. Herrero de Aza. 2020. ‘Potential Climatic Influence on Maximum Stand Carrying Capacity for 15 Mediterranean Coniferous and Broadleaf Species’. *Forest Ecology and Management* 460 (March):117824. https://doi.org/10.1016/j.foreco.2019.117824.

Rutkoski, J. E. 2019. ‘Chapter Four - A Practical Guide to Genetic Gain’. In *Advances in Agronomy*, edited by D. L. Sparks, 157:217–49. Academic Press. https://doi.org/10.1016/bs.agron.2019.05.001.

Savolainen, O., T. Pyhäjärvi, and T. Knürr. 2007. ‘Gene Flow and Local Adaptation in Trees’. *Annual Review of Ecology, Evolution, and Systematics* 38 (Volume 38, 2007): 595–619. https://doi.org/10.1146/annurev.ecolsys.38.091206.095646.

Smith, N. J. 1993. ‘Estimating Leaf Area Index and Light Extinction Coefficients in Stands of Douglas-Fir ( *Pseudotsuga* *Menziesii* )’. *Canadian Journal of Forest Research* 23 (2): 317–21. https://doi.org/10.1139/x93-043.

Tonnabel, J., T. J. M. Van Dooren, J. Midgley, P. Haccou, A. Mignot, O. Ronce, and I. Olivieri. 2012. ‘Optimal Resource Allocation in a Serotinous Non-Resprouting Plant Species under Different Fire Regimes’. *Journal of Ecology* 100 (6): 1464–74. https://doi.org/10.1111/j.1365-2745.2012.02023.x.
