# Supplementary material for: Can Thinning Foster Forest Genetic Adaptation to Drought? A Demo‐Genetic Modelling Approach With Disturbance Regimes
Source: Evol Appl. 2024 Dec 9;17(12):e70051. doi: 10.1111/eva.70051 (PMC11627118; doi:10.1111/eva.70051)
Supplement: Supplementary file 2 — Appendix S2. Calibration of three drought stress regime generators. [file EVA-17-e70051-s002.docx]

Victor Fririon, Hendrik Davi, Sylvie Oddou-Muratorio, Gauthier Ligot, François Lefèvre

**Can Thinning Foster Forest Genetic Adaptation to Drought? A Demo-Genetic Modelling Approach with Disturbance Regimes**

# Appendix S2: Calibration of three drought stress regime generators

**
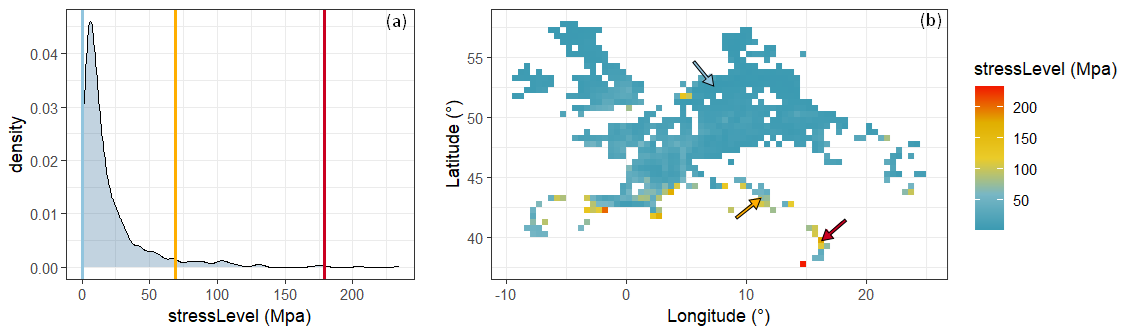
**

**Figure S1.** Average *stressLevel* over all years and LAI values within the European range of Douglas-fir: a) density plot and b) geographical distribution. The European range of Douglas-fir spans 698 raster cells, each with a resolution of 0.5° by 0.5°. The range of average *stressLevel* was wide, ranging from 0 to 234 MPa, with a median value of 11 MPa. It is noteworthy that, as a sum of daily soil water potential, *stressLevel* is inherently negative. However, constrained by the Gamma distribution framework, we used the absolute *stressLevel* values. This approach also makes the most intuitive interpretation: a higher positive *stressLevel* value corresponds to higher drought stress. Douglas-fir populations were mainly present in France, Germany, the British Isles, Italy (specifically in the Apennines), the Czech Republic, Belgium, the Netherlands and Denmark. Marginal populations were found as far as the Highlands of Scotland, the slopes of Etna in Sicily, the mountains of Portugal, the plains of Poland, and the Carpathians. Raster cells with the highest average *stressLevel* were predominantly located in Southern Europe, while the average *stressLevel* remained relatively low, or zero, in the rest of Europe.

Based on the average *stressLevel* in the raster cells, three contrasting drought stress regimes were selected. First, some raster cells showed **zero drought stress**. Then, we aimed to select the raster cell with the highest average *stressLevel*. However, both this raster cell and the next in line displayed a non-monotonic relationship between the shape and scale parameters and LAI, which did not align with the drought stress regime formalism in Luberon2 (main text, eq. 1,2). Therefore, we retained the raster cell with the third-highest average *stressLevel*, with a value of 179 Mpa, as the **severe drought stress regime**. Finally, we selected a raster cell with a **medium** drought stress regime, where the average *stressLevel* was 69 MPa. The position of these raster cells is indicated by vertical lines on the density plot and by arrows on the geographical distribution, with colours representing different stress levels: blue for zero drought stress, yellow for medium drought stress, and red for severe drought stress. The blue arrow points to one of the drought stress-free raster cells.

**Table S1.** Values of the six parameters linking the scale and shape parameters of the *stressLevel* Gamma distribution to LAI for the medium and severe drought stress regimes (main text, eq. 1,2). In the case of the zero drought stress, the drought disturbance was simply deactivated. For the medium stress drought regime, the relationship between the scale parameter and LAI displayed an R² value of 0.98, while the relationship between the shape parameter and LAI displayed an R² value of 0.99. For the severe drought stress regime, the relationship between the scale parameter and LAI displayed an R² value of 0.85, while the relationship between the shape parameter and LAI displayed an R² value of 0.92.

| **Drought stress regime** | **a_scale_** | **b_scale_** | **𝑐_scale_** | **a_sℎ𝑎𝑝𝑒_** | **b_sℎ𝑎𝑝𝑒_** | **𝑐_sℎ𝑎𝑝𝑒_** |
| --- | --- | --- | --- | --- | --- | --- |
| *Medium* | −27.9 | −0.7 | 17.9 | 1001.6 | −6.2 | 7.6 |
| *Severe* | −189.0 | −3.5 | 26.3 | 0.3 | 1.3 | 3.7 |


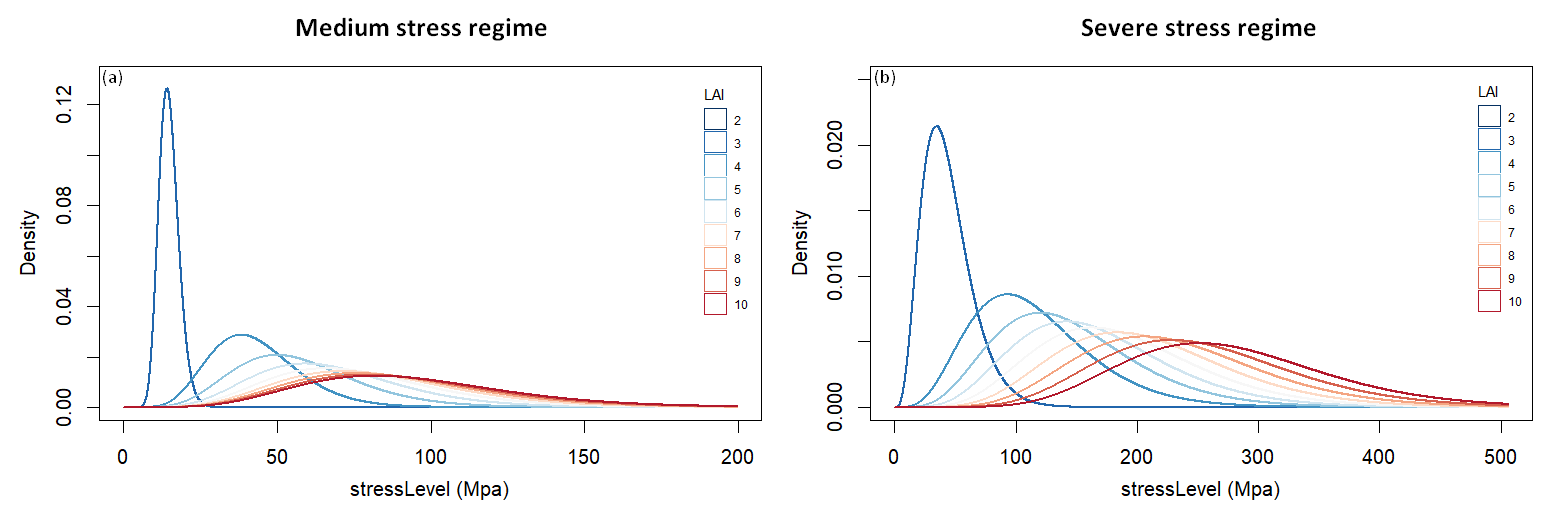


**Figure S2.** The *stressLevel* Gamma distributions modulated by LAI for the medium drought stress regime (a) and the severe drought stress regime (b), as indicated by the respective headers. For both regimes, a higher LAI is associated with an increased probability of high *stressLevel*, and it also results in higher interannual variability in *stressLevel*. Additionally, as LAI increases, the *stressLevel* Gamma distribution becomes less responsive to changes in LAI.
