# Supplementary material for: Can Thinning Foster Forest Genetic Adaptation to Drought? A Demo‐Genetic Modelling Approach With Disturbance Regimes
Source: Evol Appl. 2024 Dec 9;17(12):e70051. doi: 10.1111/eva.70051 (PMC11627118; doi:10.1111/eva.70051)
Supplement: Supplementary file 3 — Appendix S3. Supplementary figures. [file EVA-17-e70051-s004.docx]

Victor Fririon, Hendrik Davi, Sylvie Oddou-Muratorio, Gauthier Ligot, François Lefèvre

**Can Thinning Foster Forest Genetic Adaptation to Drought? A Demo-Genetic Modelling Approach with Disturbance Regimes**

# Appendix S3: Supplementary Figures


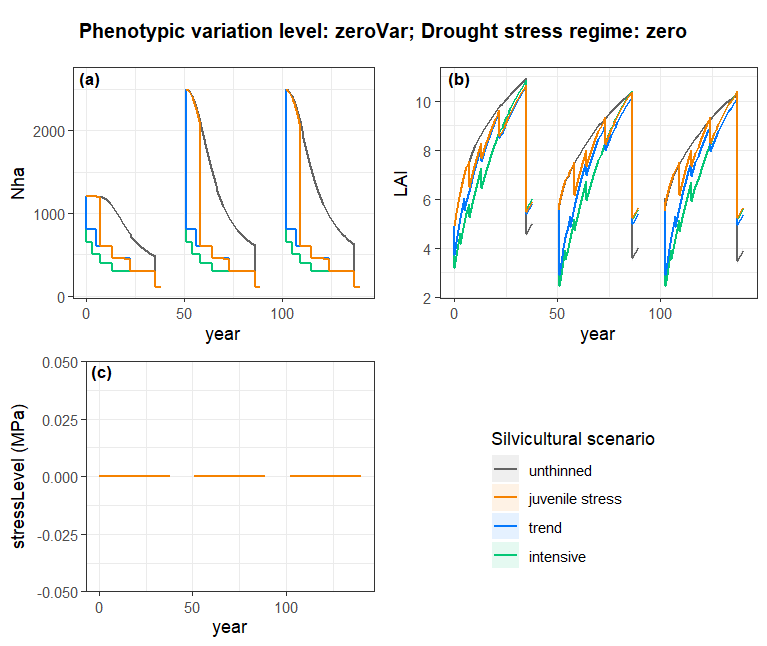


**Figure S1 (Part 1).** Characterization of stand dynamics in the four different silvicultural scenarios (colours): a) stand density, *Nha*; b) leaf area index, *LAI*; c) drought stress level (*stressLevel*). Parts 1 to 9 illustrate all the different drought stress regimes (***zero***, ***medium*** and ***severe***) and levels of phenotypic variation (***zeroVar***, ***baseVA*** and ***twiceVA***), as indicated by the respective headers. The shaded areas represent the 95% intervals over 10 replicates for each genetic setup. These intervals can be particularly wide for *stressLevel* due to the stochasticity of drought stress regimes. The pre-recruitment period is intentionally omitted from the representation, as the model calibration for tree density and growth begins from the recruitment age. Furthermore, trees that have not yet reached recruitment age are not considered in the *Nha* and the *LAI*.


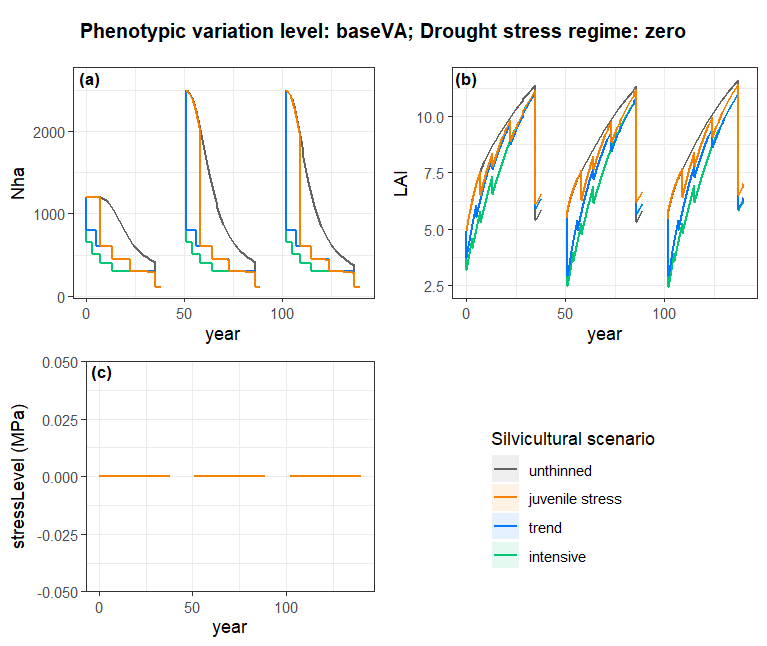


**Figure S1 (Part 2).**


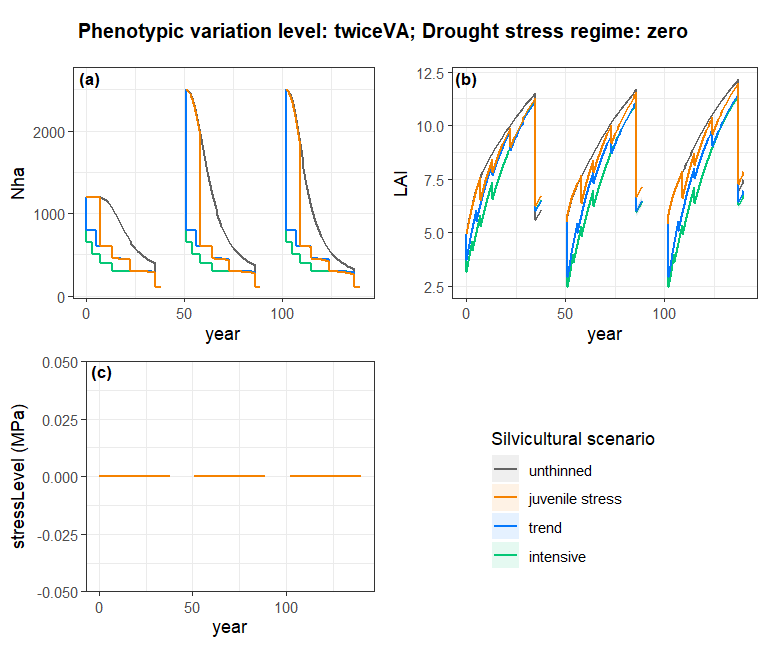


**Figure S1 (Part 3).**


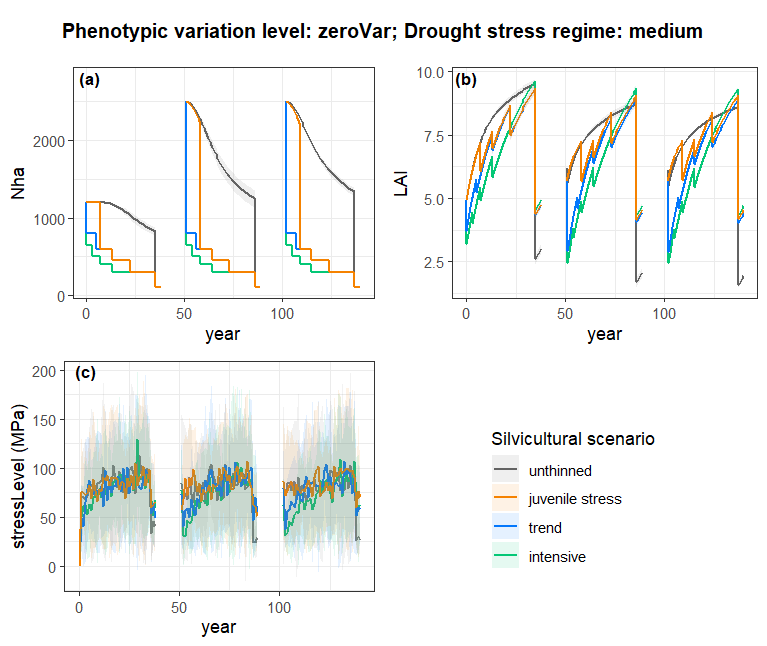


**Figure S1 (Part 4).**


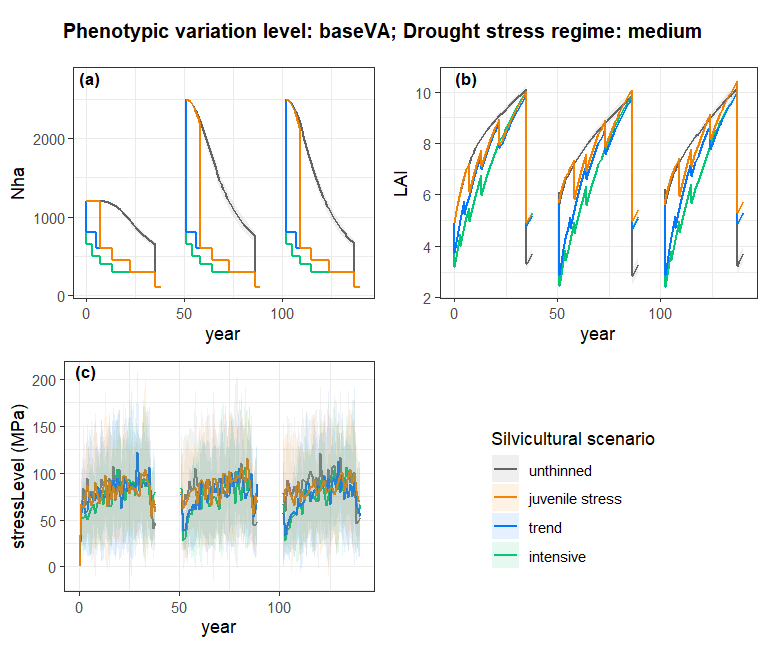


**Figure S1 (Part 5).**


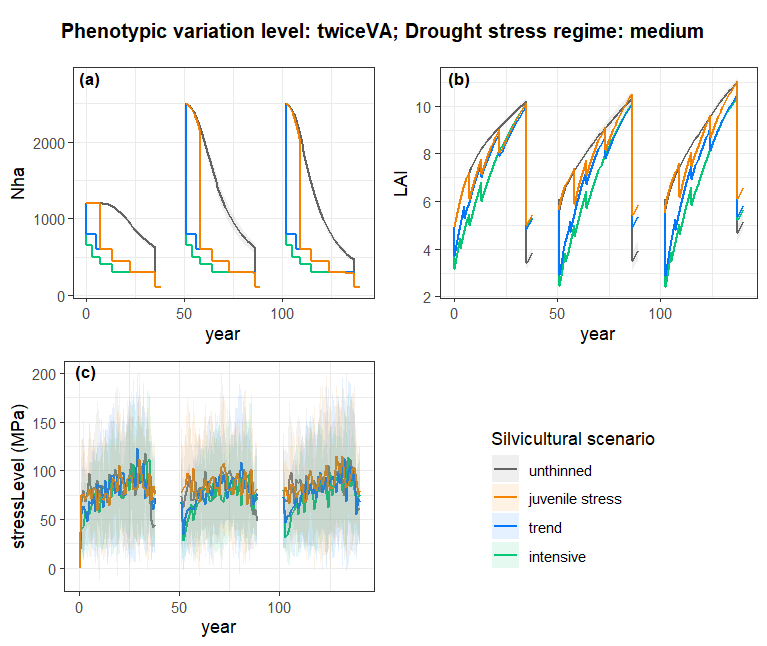


**Figure S1 (Part 6).**


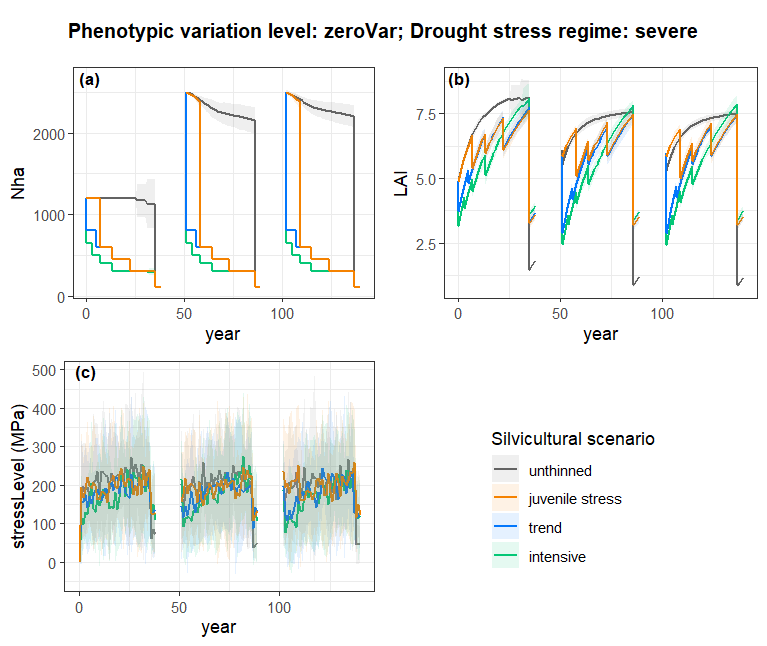


**Figure S1 (Part 7).**


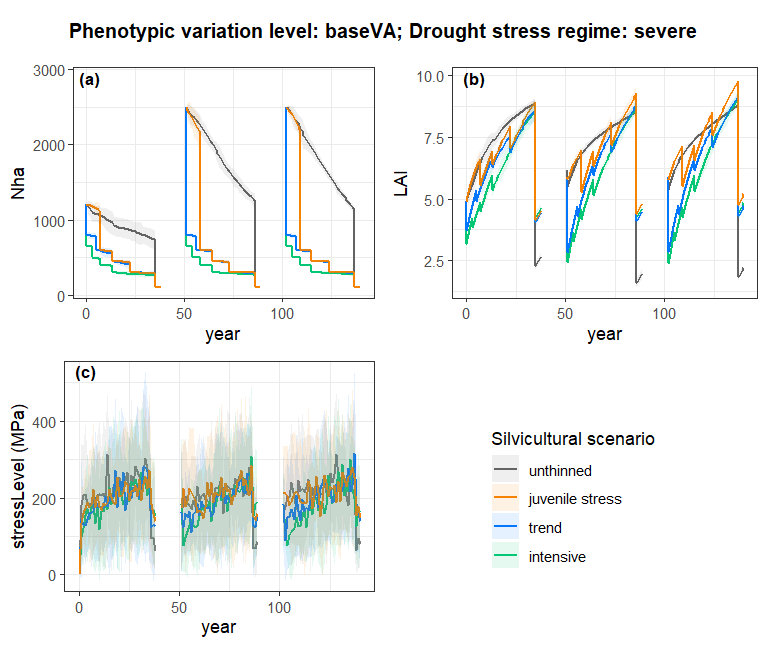


**Figure S1 (Part 8).**


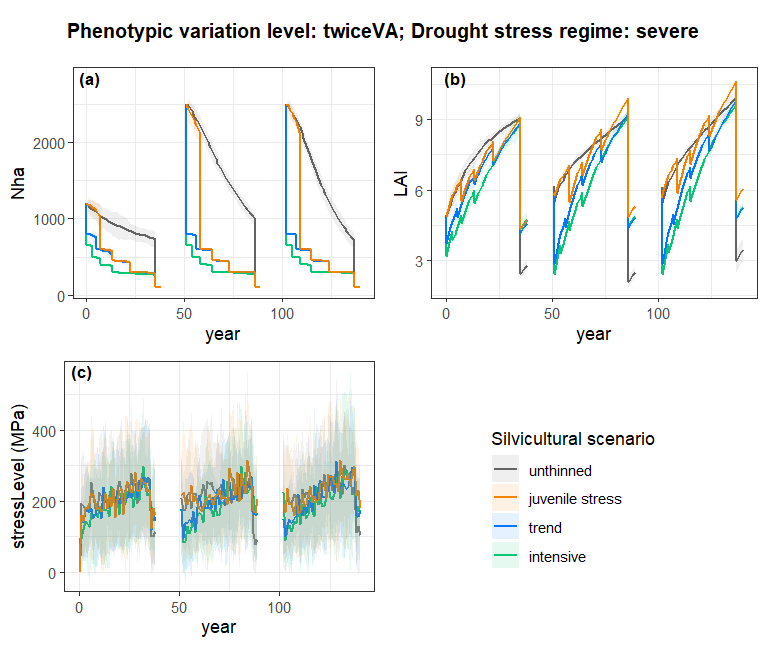


**Figure S1 (Part 9).**


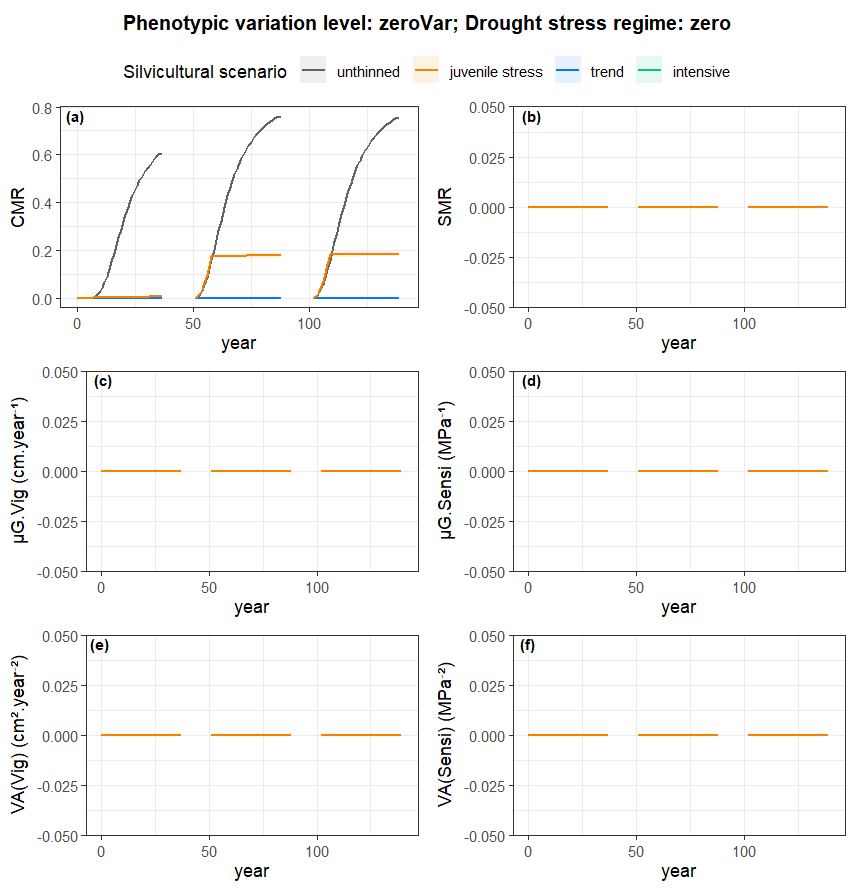


**Figure S2 (Part 1).** Dynamics of selective mortality and genetic changes across three successive rotations in four distinct silvicultural scenarios (colours): a) competition-induced mortality rate, *CMR*; b) drought stress-induced mortality rate, *SMR*; c) population genetic mean of vigour, *µG.Vig*; d) population genetic mean of sensitivity, *µG.Sensi*; e) additive genetic variance of vigour, *VA(Vig)*; f) additive genetic variance of sensitivity, *VA(Sensi)*. Parts 1 to 9 illustrate all the different drought stress regimes (***zero***, ***medium*** and ***severe***) and levels of phenotypic variation (***zeroVar***, ***baseVA*** and ***twiceVA***), as indicated by the respective headers. In the absence of drought stress, sensitivity underwent no selective pressure; any minor changes were solely a result of genetic drift. The shaded areas represent the 95% intervals over 10 replicates for each genetic setup. The pre-recruitment period is intentionally omitted from the representation, as the model does not account for any demo-genetic changes during this phase.


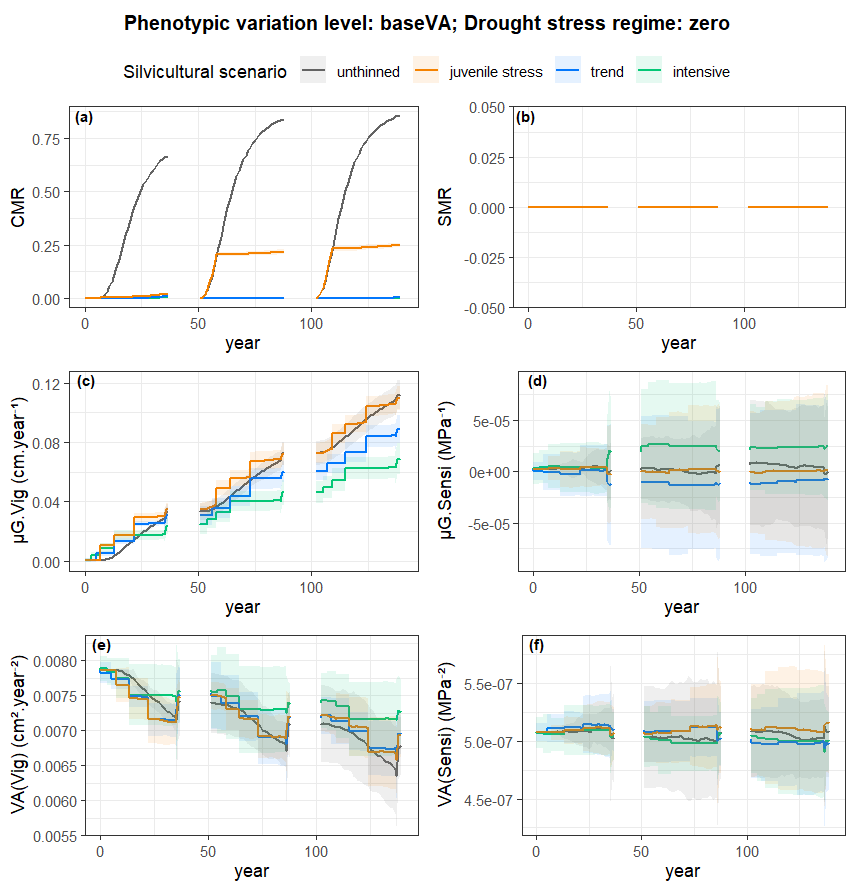


**Figure S2 (Part 2).**


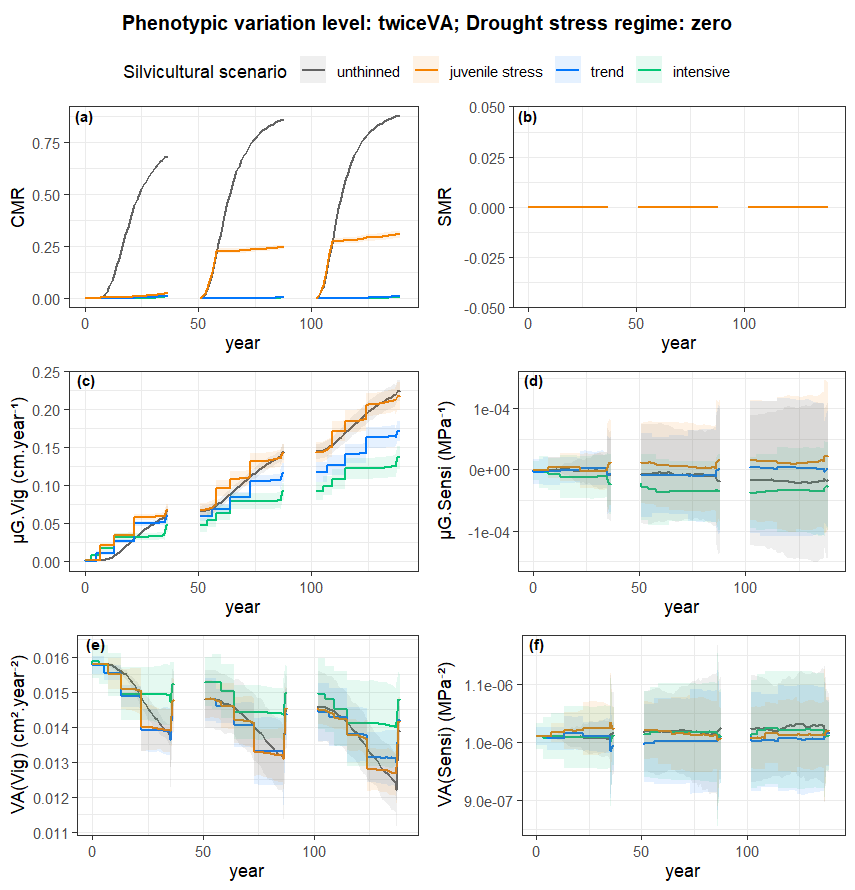


**Figure S2 (Part 3).**


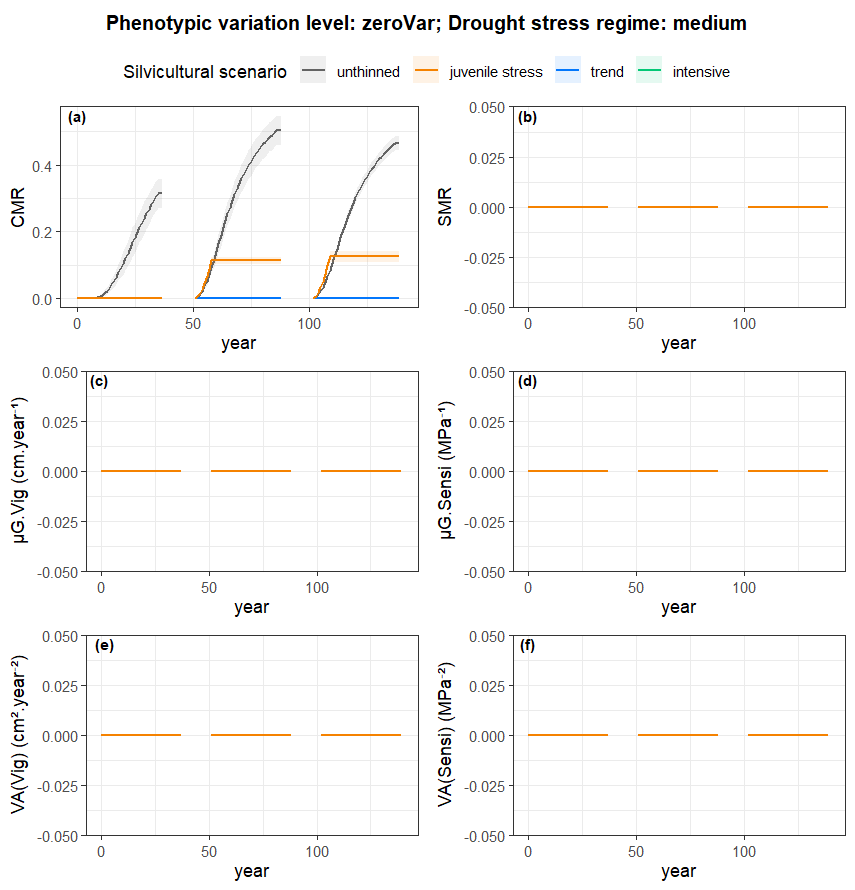


**Figure S2 (Part 4).**


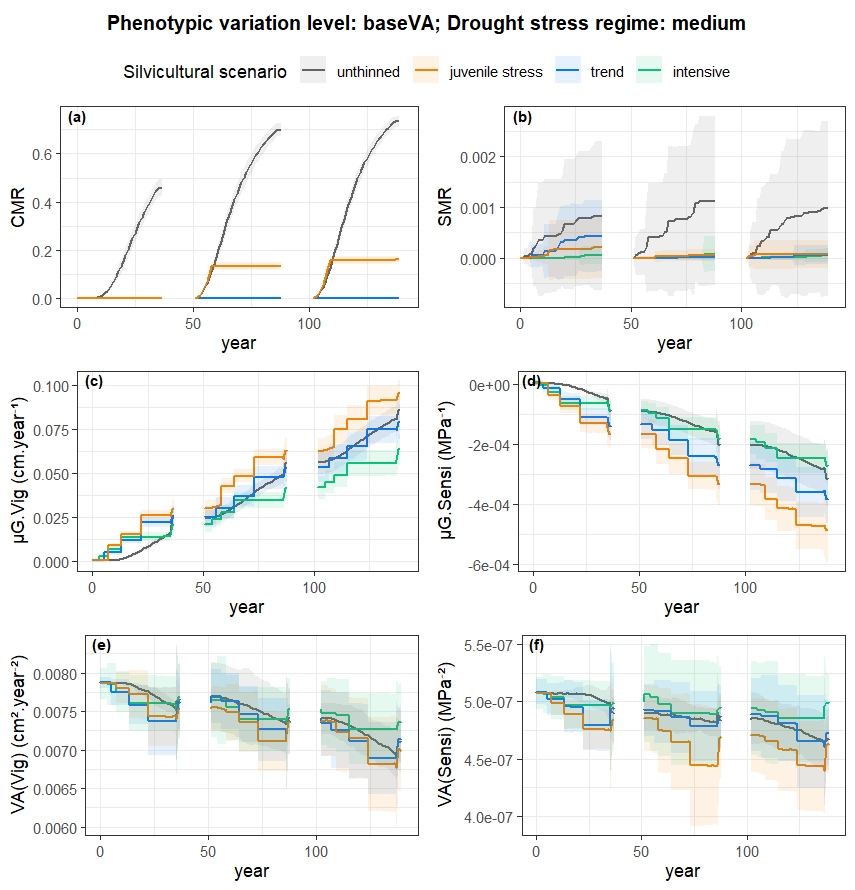


**Figure S2 (Part 5).**


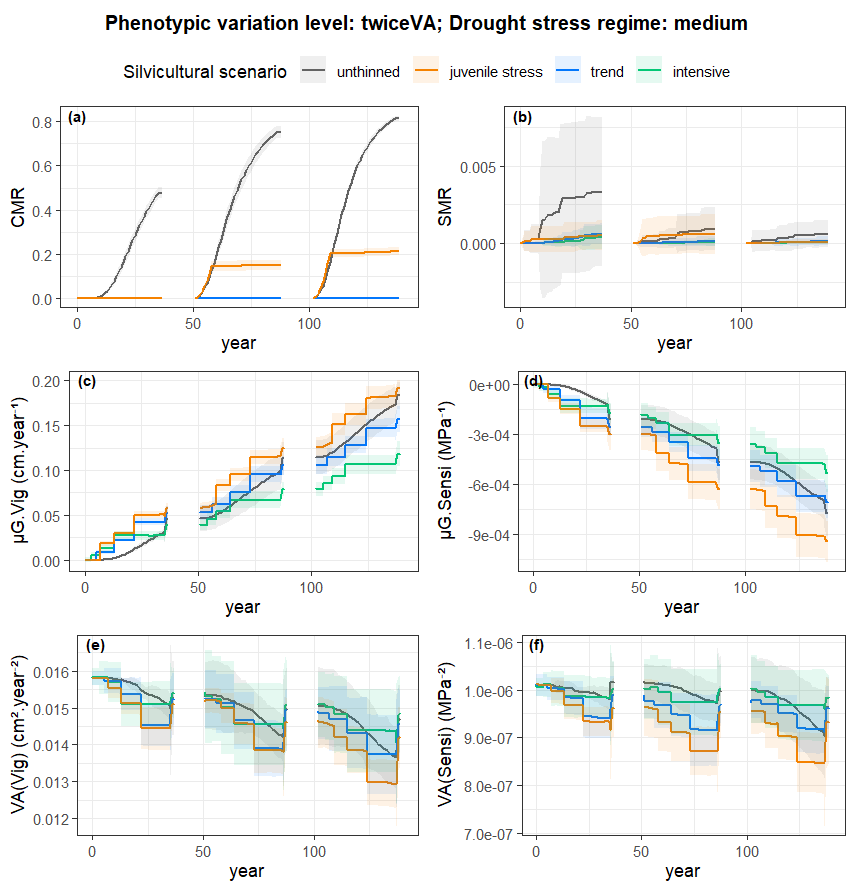


**Figure S2 (Part 6).**


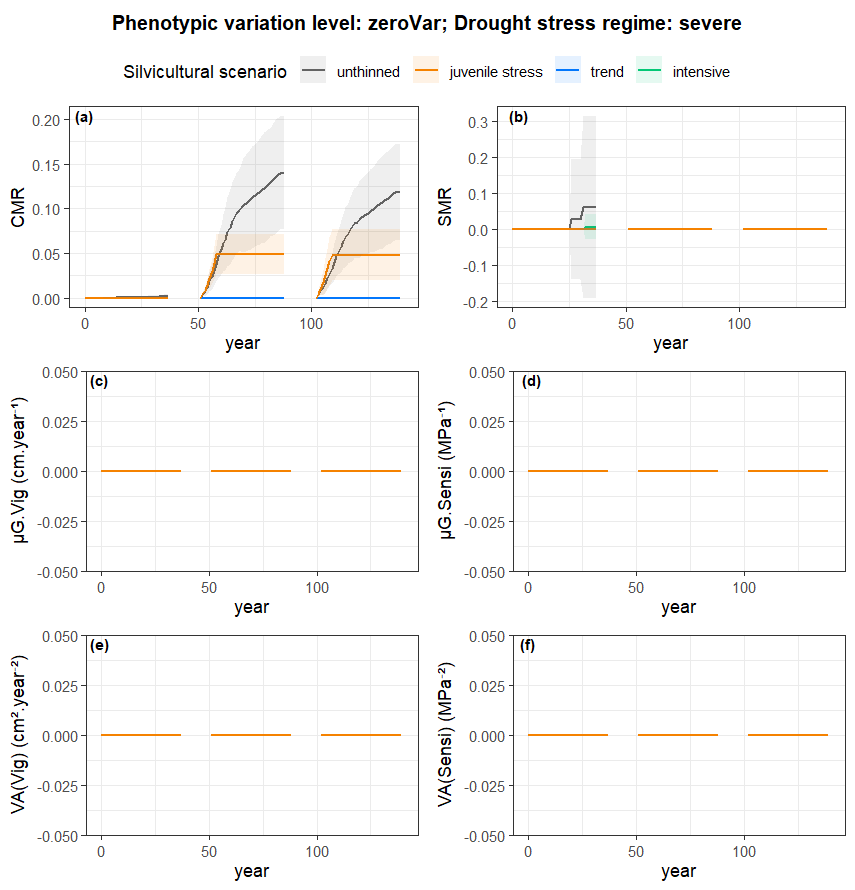


**Figure S2 (Part 7).**


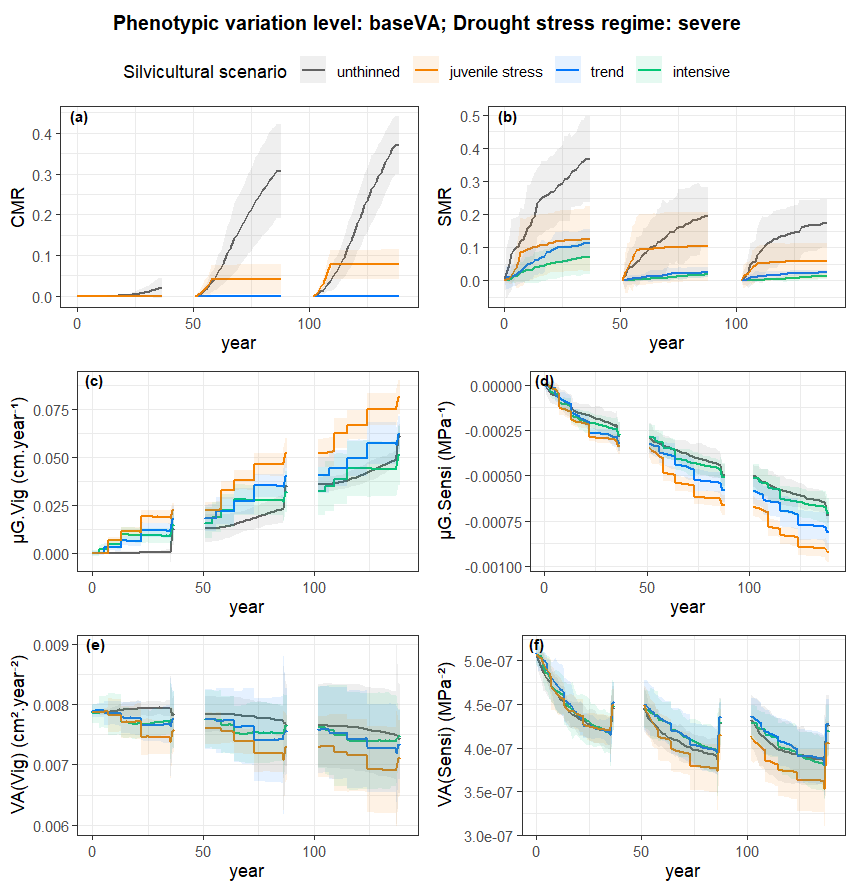


**Figure S2 (Part 8).**


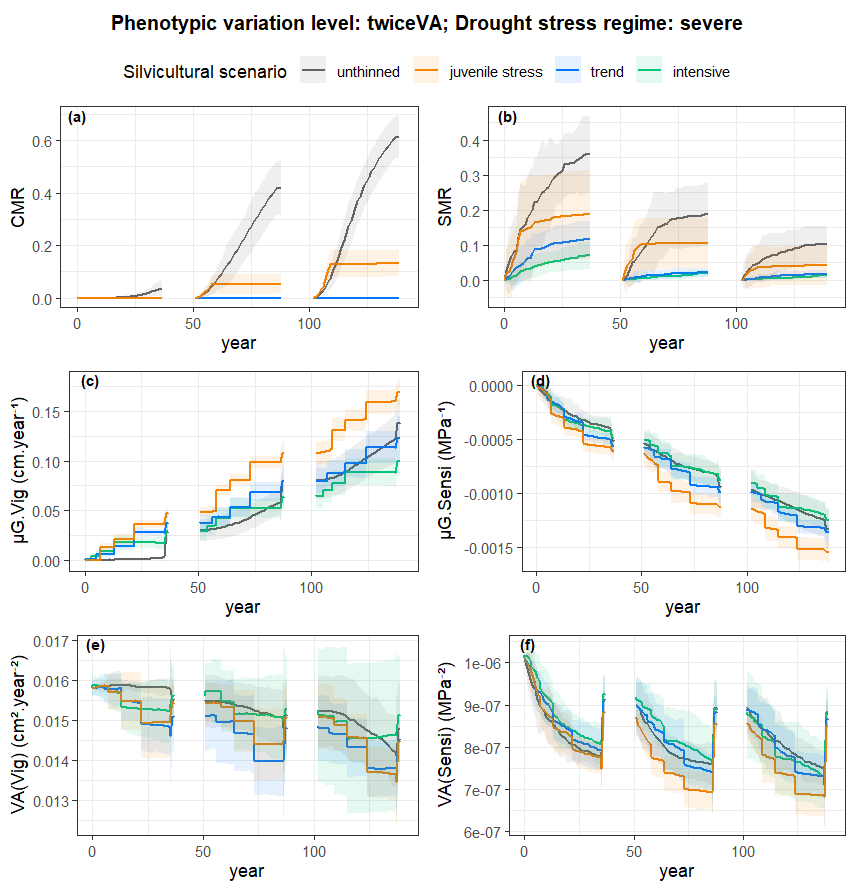


**Figure S2 (Part 9).**


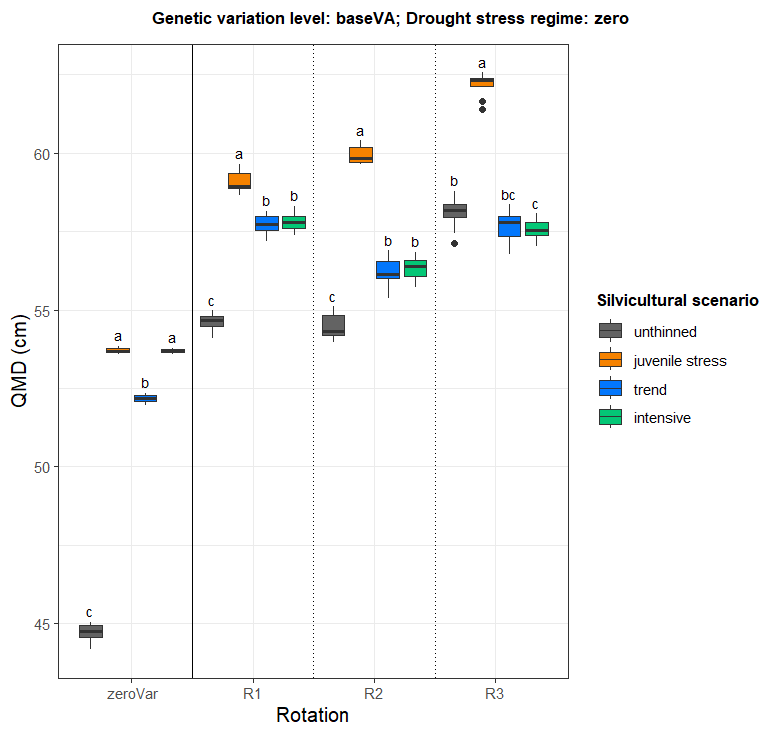


**Figure S3 (Part 1).** Quadratic mean diameter (*QMD*) at the end of three successive rotations (R1, R2 and R3) with phenotypic variation in four distinct silvicultural scenarios (colours). The end-of-rotation quadratic mean diameter without phenotypic variation (*zeroVar*) is shown as an average across three rotations and is presented as a theoretical reference in the left panel. Tukey tests were performed independently for R1, R2, R3, and *zeroVar*, with letters indicating significance between silvicultural scenarios at the 0.05 threshold. In this context, the non-significant differences arise from the stochastic components of the model, since all other conditions are identical. Parts 1 to 6 illustrate all the different drought stress regimes (***zero***, ***medium*** and ***severe***) and levels of genetic variation (***baseVA*** and ***twiceVA***), as indicated by the respective headers. The boxplots show the distribution of values obtained from 10 replicates in each case.

Without phenotypic variation, the ranking of the silvicultural scenarios in terms of quadratic mean diameter (QMD) did not constantly correspond to the anticipated plastic response. Although the first random thinning reduced competition and drought stress, thereby improving growth, it eliminated many trees regardless of size while reducing the intensity of selection on tree size. This pushed the juvenile stress scenario, which is devoid of first random thinning, up in the QMD ranking when the impact of drought stress on growth was moderate or zero: under the medium drought stress regime the juvenile stress scenario resulted in a QMD greater than the trend scenario, while, in the absence of drought stress, it resulted in a QMD equivalent to that of the intensive scenario. Otherwise, the ranking of the silvicultural scenarios in terms of tree size corresponded to the straightforward plastic response.


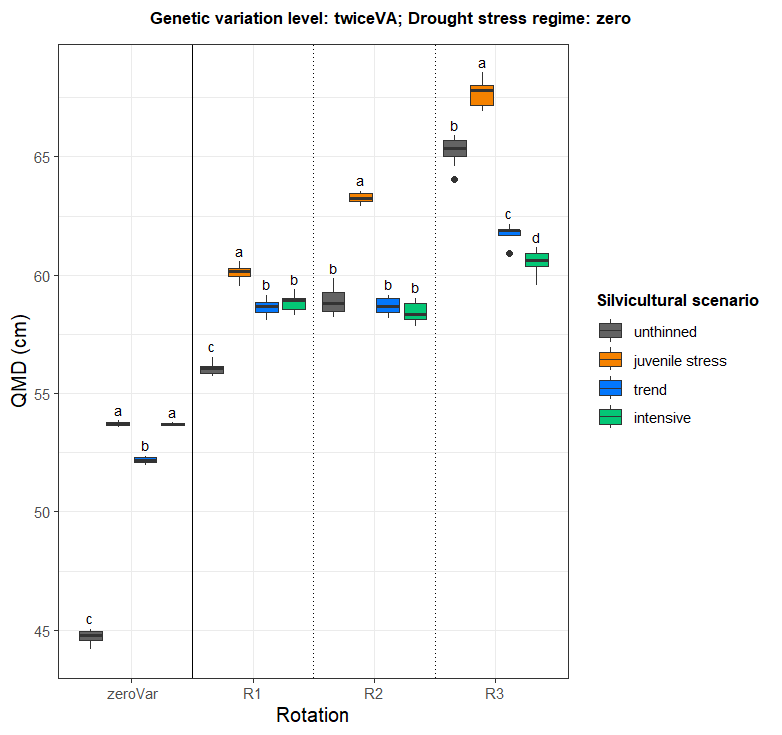


**Figure S3 (Part 2).**


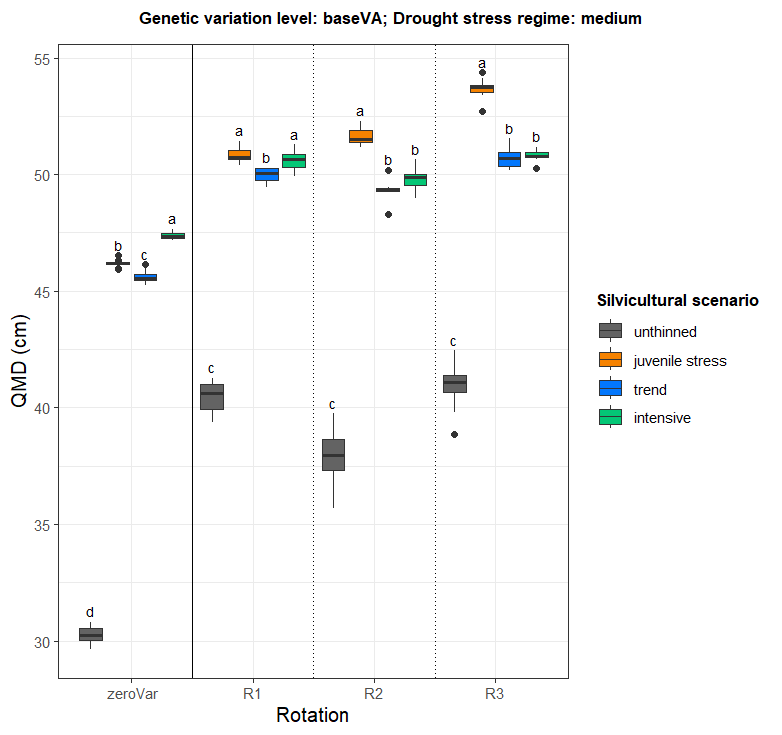


**Figure S3 (Part 3).**


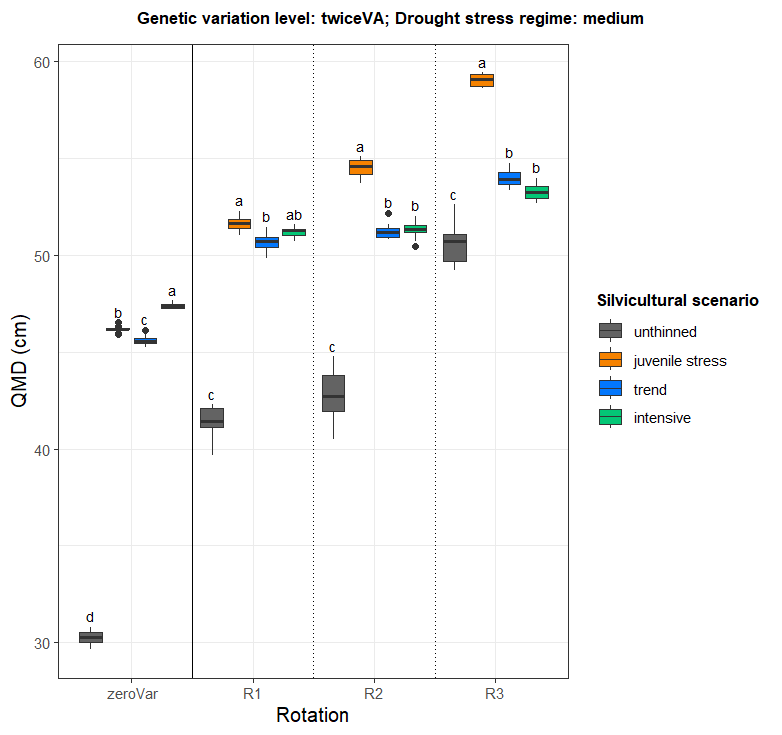


**Figure S3 (Part 4).**


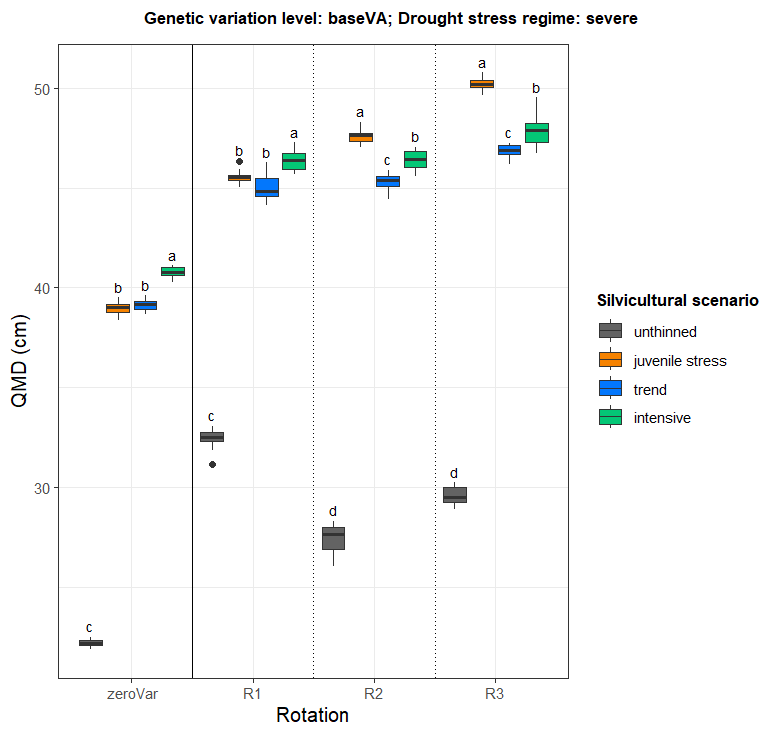


**Figure S3 (Part 5).**


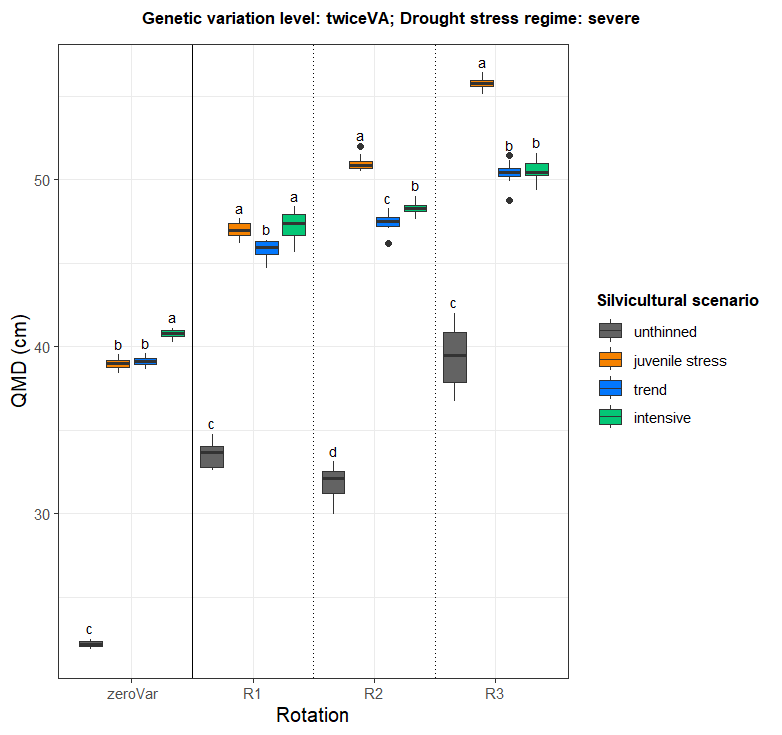


**Figure S3 (Part 6).**
