# Supplementary material for: Can Thinning Foster Forest Genetic Adaptation to Drought? A Demo‐Genetic Modelling Approach With Disturbance Regimes
Source: Evol Appl. 2024 Dec 9;17(12):e70051. doi: 10.1111/eva.70051 (PMC11627118; doi:10.1111/eva.70051)
Supplement: Supplementary file 4 — Appendix S4. Correcting the impacts of phenotypic variation in vigour on growth prediction by a demo‐genetic coupled model. [file EVA-17-e70051-s001.docx]

Victor Fririon, Hendrik Davi, Sylvie Oddou-Muratorio, Gauthier Ligot, François Lefèvre

**Can Thinning Foster Forest Genetic Adaptation to Drought? A Demo-Genetic Modelling Approach with Disturbance Regimes**

# Appendix S4: Correcting the impacts of phenotypic variation in vigour on growth prediction by a demo-genetic coupled model

## Summary

All trees in a forest have different growth capacities driven by their unique genotype and microenvironment, which combines the microsite conditions and the individual social status dynamically driven by stand dynamics. This phenotypic variability results in individual variability of achieved growth around the mean, which has a feedback consequence on stand growth because it interferes with asymmetric competition, reproduction and selective mortality processes. Many growth models do not explicitly consider all terms of the phenotypic variability, apart from the social status. More precisely, tree variability often does not appear as such in the growth equations. However, the empirical data used to parameterize these equations integrate its consequence on observed stand growth. In other words, the consequence of phenotypic variability implicitly hides in the parameterization of such models, without appearing explicitly in the equations.

The demo-genetic model *Luberon2* results from the introduction of a phenotypic effect in the growth equations derived from a baseline growth model that originally did not explicitly consider the phenotypic effect. In a previous publication (Godineau, Fririon et al, 2023, appendix S4), we analysed how the addition of phenotypic vigour changes the baseline prediction of individual tree growth and, therefore, deviates from the observed data used for the calibration of the baseline growth model. Indeed, adding an explicit phenotypic effect in the demo-genetic model without changing the original parameterization of the growth model results in over-accounting for the phenotypic effect, both explicitly and implicitly in the original parameterization.

Here, we first analyse the mechanisms through which the introduction of phenotypic variation in vigour progressively leads to over-estimate individual tree growth. Then, we propose a correction of the causal mechanisms of this bias, which consists in re-adjusting the growth model parameterization at each simulation step in order to stick to appropriate growth prediction after including the explicit phenotypic effect. We show that this correction is efficient: the predicted growths with correction fit the original baseline predictions, i.e. they fit the empirical data used for calibration of the baseline growth model. We also show that the bias on growth prediction without this correction has negligible consequences on the estimate of the evolutionary rates, i.e. the evolutionary rates predicted with or without the correction are very similar, after a few generations. This is because, at this time frame, the main driver of evolution is the relative ranking of tree dimensions rather than their absolute size. Activating the correction is optional. We recommend enabling it for forecasts of growth and population dynamics consistent with empirical data. For exploring evolutionary processes and selection dynamics, especially under disturbance regimes, the correction should be turned off.

## Introduction

*Luberon2* builds upon a distance-independent tree growth model, which predicts the baseline radial increment for each tree at every annual step based on its initial girth (**Fig. S1**; Deleuze et al., 2004 for more details). The parameters of this relationship, i.e. girth threshold and slope, vary dynamically depending on dendrometric variables (e.g., dominant height, basal area, stand density). The dendrometric variables that drive the growth-girth relationship may vary among species. Originally, the baseline growth model, calibrated on empirical data, does not account for individual effects in its equations.


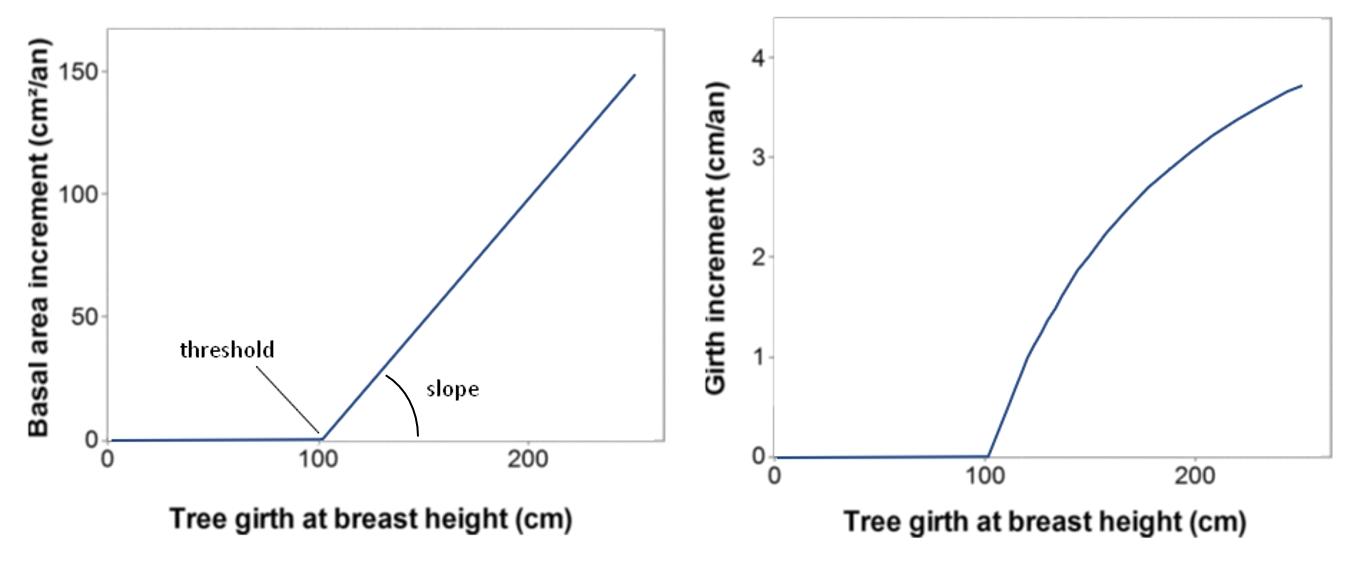


***Figure S1.*** *Baseline prediction of the annual individual radial increment as a function of initial girth. The left panel represents tree basal area increment (cm^2^/year); the right one represents tree girth increment (cm/year). The relationship between tree basal area increment and girth is determined by two growth parameters: the girth threshold and the slope. Girth increment is directly derived from tree basal area increment and girth, considering the geometric effect. This figure is taken from Perin et al. 2017. In* Luberon2*, this baseline growth model has minor detail variations depending on the species.*

In a previous study (Godineau, Fririon et al., 2023), we conducted an analysis (Appendix 4) to assess how introducing interindividual phenotypic variation in vigour alters the baseline prediction of annual tree growth and tree diameter throughout stand development, both within and between generations. For a given tree, vigour is defined as the individual deviation (positive or negative) from the baseline prediction of the growth model: a tree can grow better or worse than the average prediction of the baseline model due to its phenotype. At initialization, the population mean of vigour is generally set to 0: individual vigour values are centred around the predictions of the growth model. Furthermore, girth and genetic values are assigned independently, resulting in a zero correlation between the two at initialization.

Intuitively, the problem can be formulated as follows: in the raw demo-genetic coupled model (with individual variation before any correction), the predicted growth of bigger trees deviates more from the prediction of the baseline growth model, and therefore from empirical data, which can ultimately result in over-predicted mean stand growth once selective self-thinning occurs.

In this document, we aim to deepen the understanding of the nature and dynamics of the deviation from the baseline growth model within generations in order to develop an appropriate correction of the demo-genetic coupled model. The solution we propose implements a dynamic correction to the growth functions of the model.

## Deviation from the baseline growth model in the raw demo-genetic coupled model

### … before self-thinning

On average, adding individual variation in vigour does not change annual growth before self-thinning (population mean of vigour is 0). Yet larger trees exhibit excess growth relative to baseline predictions, while smaller trees exhibit diminished growth. This arises from the gradual establishment of a positive correlation between vigour and girth, as vigorous trees exhibit faster growth while less vigorous ones grow slower (**Fig. S2**). Changes in the hierarchy of girth are primarily limited to the early annual steps and are solely attributed to the disconnection between initial girth and vigour values. Overall, adding individual variation in vigour results in an average deviation from the baseline growth model, which depends linearly on girth.


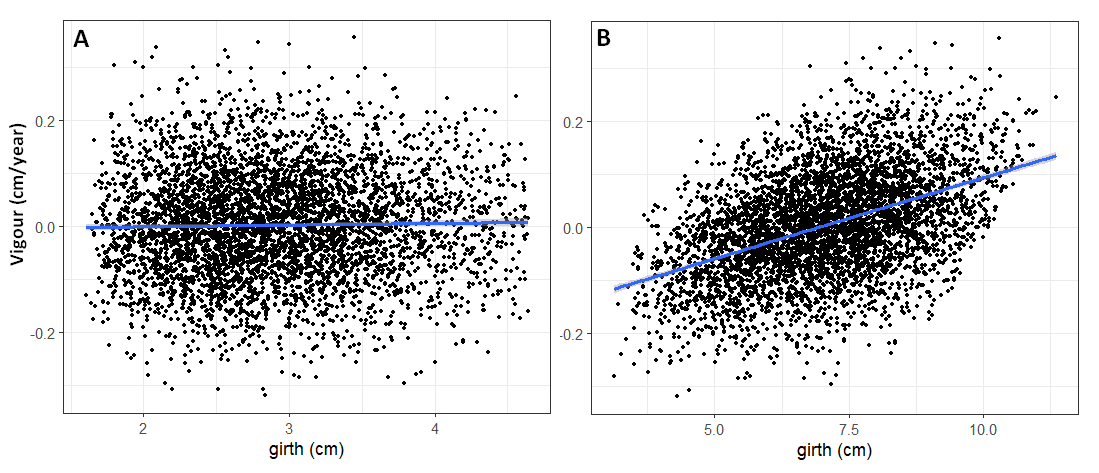


***Figure S2.*** *Relationship between vigour (phenotypic values) and girth before self-thinning: at initialization (A) and after 15 annual steps (B). Each point represents a tree characterised by its girth and vigour. In both cases, the vigour distribution is centred around 0, indicating that average growth at the stand scale remains unaffected by the introduction of individual variation in vigour. At initialization (A), the correlation between vigour and girth is zero. After 30 years (B), the positive relationship between vigour and girth implies that, on average, that large trees grow excessively while small trees grow insufficiently compared to the baseline predictions. The blue lines represent the regression lines.* *The simulated species is Douglas-fir. The simulation began at age 15, the recruitment age for Douglas-fir in Luberon2.*

In addition, as a runaway process, the further a tree deviates from the baseline growth model, the more its girth progressively diverges from the reference without variation. Thus, the radial increment of a big tree predicted by the raw demo-genetic coupled models, computed from its initial girth, deviates even further from the prediction of the baseline growth model.

### … after self-thinning

When self-thinning begins, it eliminates smaller, less vigorous trees within the vigour-girth relationship (**Fig. S3**). As a result, adding individual variations in vigour changes the predicted mean annual growth and mean diameter.


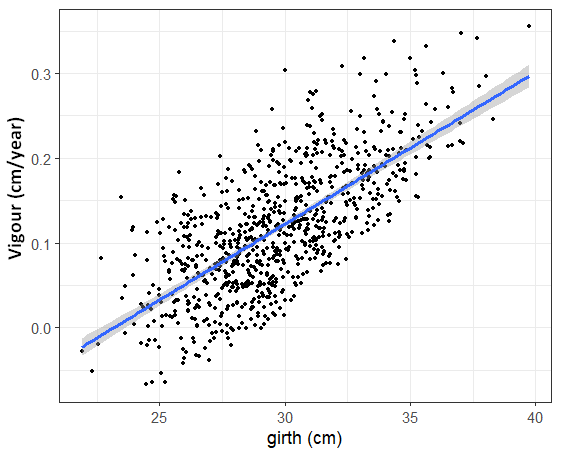


***Figure S3.*** *Relationship between vigour (phenotypic values) and girth after self-thinning (85 annual steps after initialization). Each point represents a tree characterised by its girth and vigour. The vast majority of surviving trees has positive vigour. The deviation from the baseline growth model still depends on girth. The blue line represents the regression line. The simulated species is Douglas-fir. The simulation began at age 15, the recruitment age for Douglas-fir in Luberon2.*

In addition, the parameters of the baseline growth model depend on dendrometric variables which are affected by the demo-genetic coupling (e.g., stand density, basal area). Thus, the demo-genetic coupling directly affects these growth parameters. This holds true for all *Luberon2* species, albeit not necessarily on the same parameter(s) or with the same sensitivity (**Fig. S4** illustrates the case of cedar).


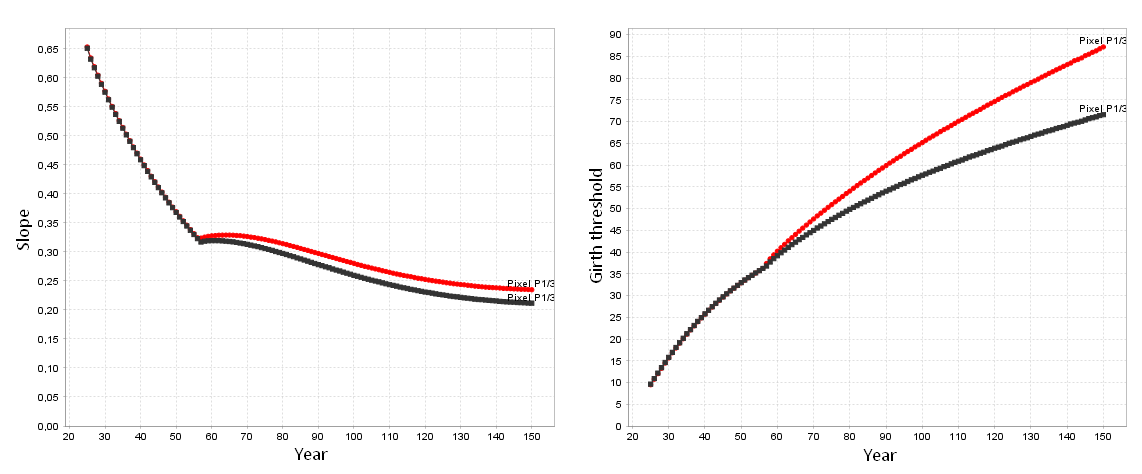


***Figure S4.*** *Dynamics of the two parameters of the baseline growth model for cedar: slope on the left and girth threshold on the right. Results from the baseline growth model are represented in black, those from the raw demo-genetic coupled model in red. For cedar, the girth threshold and slope depend on two dendrometric variables: the sum of tree diameters and the number of trees per hectare. The divergence between model parameters starts from self-thinning, that is to say when the dendrometric variables begin to diverge.*

## Choosing an appropriate correction

We sought to correct the average deviation from the baseline growth model predictions, which is determined by the (positive) relationship between vigour and girth. At each annual step, the corrected predicted tree growth is computed through the following steps (**Fig. S5**):

- The baseline growth model computes the baseline growth predictions in terms of girth increment (see **Fig. S1**);
- a linear regression (*Vigour = β_0_ + β_1_ × Girth*) is used to estimate the predicted values of the vigour-girth relationship (see **Fig. S2,S3**);
- the baseline growth predictions are then corrected by subtracting the predicted values;
- to preserve genetic gain from one generation to the next, which would otherwise be annihilated by the preceding step, the initial genetic mean of the population is added (“initial” refers to either simulation initialization or recruitment);
- individual phenotypic vigour is added to the corrected growth predictions.

The correction assumes that the data used to calibrate the baseline growth model integrates the dynamic of the vigour-girth correlation and the selection of large vigorous trees. Thus, the correction aims to remove the impact of these two processes on the predictions of the baseline growth model, because these processes directly result from the demo-genetic dynamics of the model when adding individual variation.


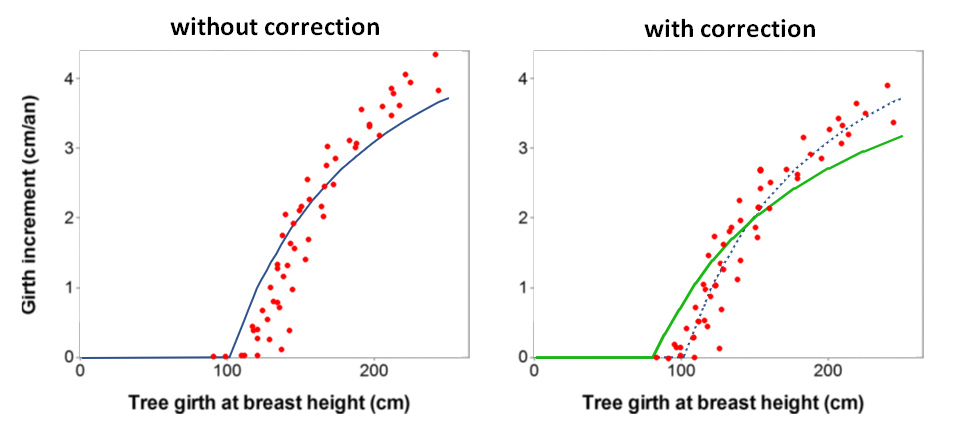


***Figure S5.*** *Diagram representing the principle of the proposed correction.* *Without correction (left): the solid blue curve represents the baseline growth model predictions in terms of girth increment. The red dots represent raw tree growth predictions (baseline growth model + individual vigour). The deviation from the baseline growth model varies linearly with girth: here, larger trees show excessive growth (positive vigour), while smaller trees show reduced growth (negative vigour). With correction (right): the dotted blue curve represents the baseline growth predictions. The solid green line represents the corrected growth model predictions, which are the baseline growth predictions minus the predicted values from the linear vigour-girth relationship. The red dots represent corrected predicted tree growth (corrected growth model + individual vigour), centred on predictions without correction: on average, there is no deviation.*

## Correction results

The results presented in this section are based on a Douglas-fir stand with an initial density of 1150 trees per hectare with a site index of 30 metres at 50 years. The simulations begin at age 15, i.e. the recruitment age for Douglas-fir in *Luberon2*, and end at age 100, i.e. 85 annual steps later. There is no thinning. The correction has been tested for all species of *Luberon2* in varied conditions, and this specific case is highly representative of all the others. Given the stochastic nature of demo-genetic processes in *Luberon2*, it is expected that minor quantitative discrepancies may arise when replicating these results.

### Effects on growth over a generation

The correction gives very satisfactory outcomes for both average growth and the growth of dominant trees, defined as the 100 largest trees per hectare (**Fig. S6**). Under the tested conditions, compared to the baseline model, the raw demo-genetic coupled model led to a 17% increase in the quadratic mean diameter and a 20% increase in the dominant diameter at age 100. Following correction, these increases were reduced to less than 1% and less than 2%, respectively. Additionally, while the raw demo-genetic coupled model led to a 37% increase in maximum diameter at age 100, this increase was reduced to 9% after correction (**Fig. S7**). Consequently, the correction also effectively mitigates deviations in the growth model parameters (not shown).


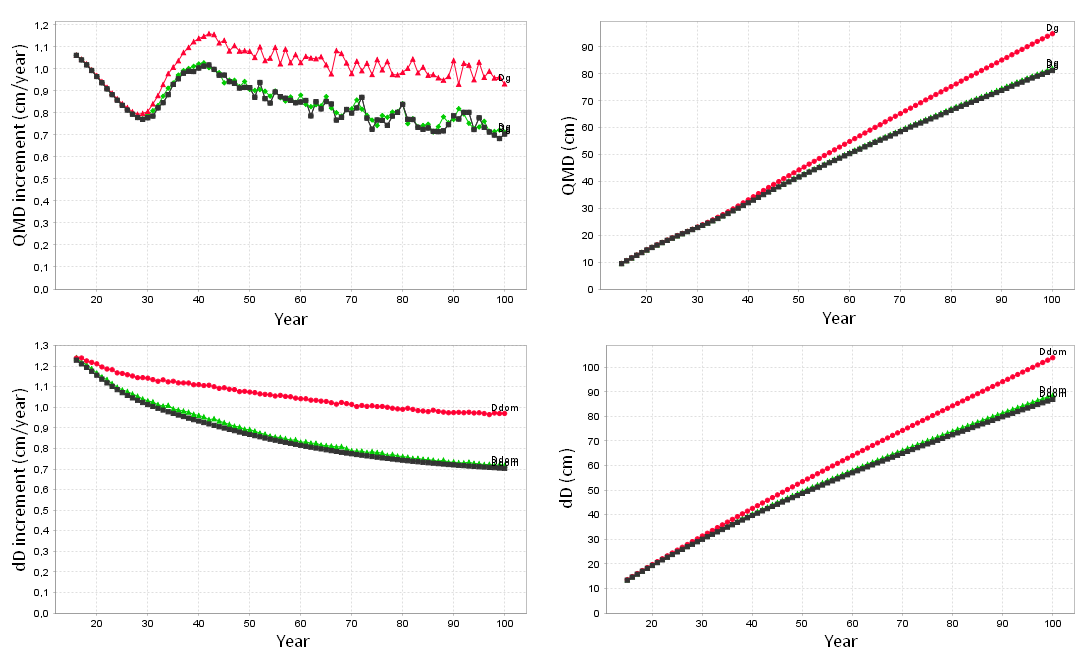


***Figure S6.*** *From top left to bottom right: quadratic mean diameter increment, quadratic mean diameter,* *dominant diameter increment, dominant diameter. Results from the baseline growth model are represented in black, those from the raw demo-genetic coupled model (without correction) in red, and those from the corrected demo-genetic coupled model in green. The black and green curves can overlap heavily, illustrating the effectiveness of the correction.*


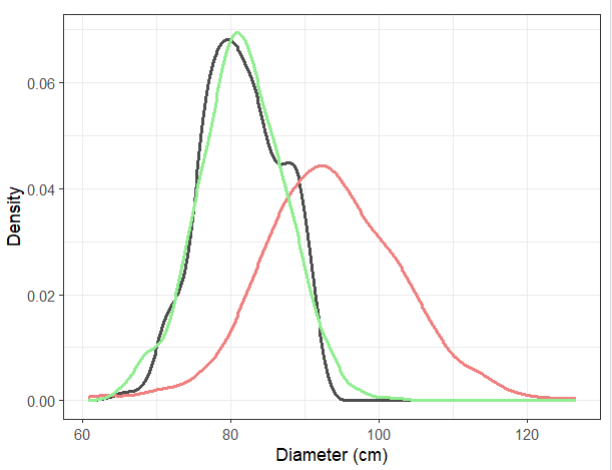


***Figure S7.*** *Density curve of diameters after 85 years of simulation. Results from the baseline growth model are represented in black, those from the raw demo-genetic coupled model (without correction) in red, and those from the corrected demo-genetic coupled model in green.*

### Effects on selection over a generation

The raw demo-genetic coupled model led to an increase in the level of competition, leading to an increase in selective self-thinning. With correction, this bias is almost entirely eliminated. The correction of growth prediction does not change the estimation of genetic changes much (**Fig. S8**). Indeed, the relative ranking of tree dimensions, the primary driver of evolution, is not affected.


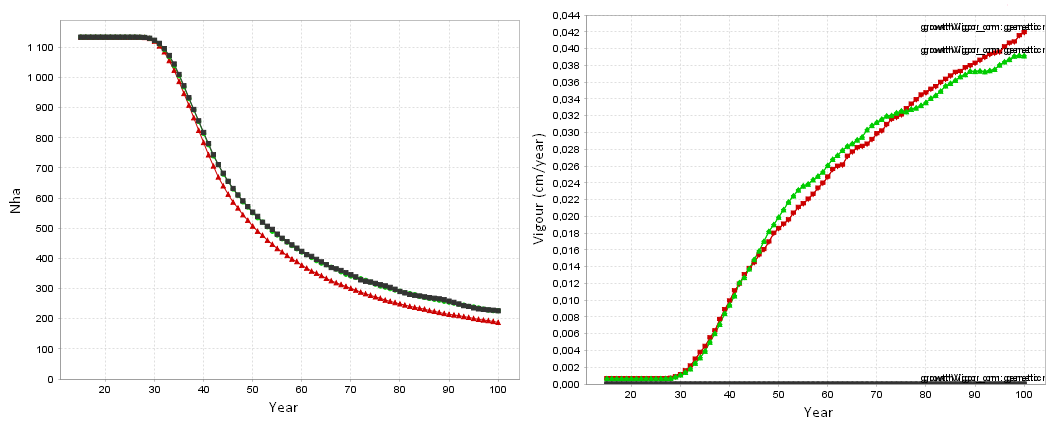


***Figure S8.*** *The number of trees per hectare on the left and the genetic gain in vigour on the right. The only cause of mortality is selective self-thinning. Results from the baseline growth model are represented in black, those from the raw demo-genetic coupled model (without correction) in red, and those from the corrected demo-genetic coupled model in green. The black and green curves can overlap heavily, illustrating the effectiveness of the correction.*

### Results over three generations

As observed previously, in the first generation, the corrected growth and mortality predictions approach those without variation (baseline). Over subsequent generations, we observe the impact of genetic improvement in vigour (**Fig. S9**): increased growth rates, leading to increased diameter, competition level, and selective self-thinning. Slightly lower growth rates are predicted with correction than without over the three generations.


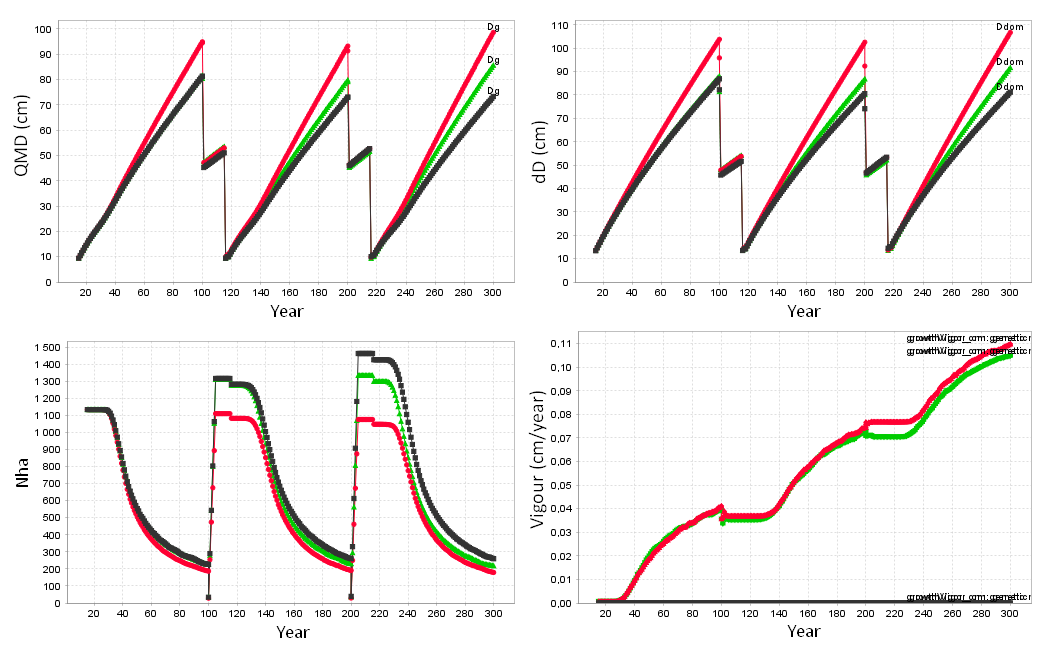


***Figure S9.*** *From top left to bottom right: quadratic mean diameter, dominant diameter, number of trees per hectare and genetic gain in vigour. Results from the baseline growth model are represented in black, those from the raw demo-genetic coupled model (without correction) in red, and those from the corrected demo-genetic coupled model in green. The black and green curves can overlap heavily, especially in the first generation. At the end of each generation, at age 100, a thinning triggers 5 years of regeneration.*

## A user choice

Whether or not to activate the correction is a user choice. Note that the correction has a negligible impact on the calculation time. To choose to activate or deactivate the correction, the objectives of the simulations and the potential impact on tree growth and evolution predictions must be considered.

The correction should be activated when priority is given to the accuracy of forest growth predictions and estimates of dendrometric variables. The correction ensures that growth predictions closely match the values expected in similar conditions as the empirical data used for the original calibration of the baseline growth model. It also ensures the accuracy of estimates for key dendrometric variables, such as quadratic mean diameter, dominant diameter, basal area, or associated allometric variables, such as leaf area. This choice can be relevant for ecological studies and practical applications such as forest management and economic planning.

The correction should be deactivated when the primary goal is to decipher evolutionary processes and eco-evolutionary feedback effects. Deactivating the correction ensures that the growth equations remain strictly identical without and with phenotypic variation, and comparing these two scenarios allows for a quantitative assessment of the interplay between ecological dynamics and evolutionary processes, both within and across generations. Conversely, when the correction is activated, the comparison incorporates differences in the growth equations themselves. Notably, over a single generation, forest dynamics will be identical regardless of whether evolutionary processes occur, rendering comparisons meaningless at this time scale. Furthermore, when comparing different disturbance regimes, applying the correction tends to constrain the dynamic variations in growth potential, thereby masking the effects of genetic changes specific to each regime. This also introduces a bias by homogenising selection pressures across the disturbance regimes.

In conclusion, the choice of whether or not to activate the correction depends on the main objective of the study. For accurate predictions of stand growth and dynamics, consistent with empirical data, the correction should be enabled. Conversely, for in-depth exploration of evolutionary processes and selection dynamics, particularly in contexts of disturbance regimes, the correction should be turned off. Additionally, as demonstrated in this appendix on the scale of a few generations—specifically, following the recommendations for using *Luberon2*—the impact of the correction on evolutionary rate predictions is negligible. However, for simulation settings that differ significantly from those presented here (e.g., involving more generations), we recommend to check beforehand that the choice made on the correction does not affect the predicted evolutionary rates.

## References

Deleuze, C., O. Pain, J.-F. Dhôte, and J.-C. Hervé. 2004. ‘A Flexible Radial Increment Model for Individual Trees in Pure Even-Aged Stands’. *Annals of Forest Science* 61 (4): 327–35. https://doi.org/10.1051/forest:2004026.

Godineau, C., V. Fririon, N. Beudez, F. de Coligny, F. Courbet, G. Ligot, S. Oddou-Muratorio, L. Sanchez, and F. Lefèvre. 2023. ‘A Demo-Genetic Model Shows How Silviculture Reduces Natural Density-Dependent Selection in Tree Populations’. *Evolutionary Applications* 16 (11): 1830–44. https://doi.org/10.1111/eva.13606.

Perin, J., H. Claessens, P. Lejeune, Y. Brostaux, and J. Hébert. 2017. ‘Distance-Independent Tree Basal Area Growth Models for Norway Spruce, Douglas-Fir and Japanese Larch in Southern Belgium’. *European Journal of Forest Research* 136 (2): 193–204. https://doi.org/10.1007/s10342-016-1019-y.
